# Fossil gaps inferred from phylogenies alter the apparent nature of diversification in dragonflies and their relatives

**DOI:** 10.1186/1471-2148-11-252

**Published:** 2011-09-14

**Authors:** Robert B Davis, David B Nicholson, Emily LR Saunders, Peter J Mayhew

**Affiliations:** 1 Department of Biology, University of York, York, YO10 5YW, UK; 2 Department of Zoology, University of Tartu, Vanemuise 46, EE-51014 Tartu, Estonia; 3 Department of Palaeontology, The Natural History Museum, Cromwell Road, London, SW7 5BD, UK; 4 National Museums of Scotland, Department of Natural Sciences, Edinburgh, Midlothian, EH1 1JF, UK

## Abstract

**Background:**

The fossil record has suggested that clade growth may differ in marine and terrestrial taxa, supporting equilibrial models in the former and expansionist models in the latter. However, incomplete sampling may bias findings based on fossil data alone. To attempt to correct for such bias, we assemble phylogenetic supertrees on one of the oldest clades of insects, the Odonatoidea (dragonflies, damselflies and their extinct relatives), using MRP and MRC. We use the trees to determine when, and in what clades, changes in taxonomic richness have occurred. We then test whether equilibrial or expansionist models are supported by fossil data alone, and whether findings differ when phylogenetic information is used to infer gaps in the fossil record.

**Results:**

There is broad agreement in family-level relationships between both supertrees, though with some uncertainty along the backbone of the tree regarding dragonflies (Anisoptera). "Anisozygoptera" are shown to be paraphyletic when fossil information is taken into account. In both trees, decreases in net diversification are associated with species-poor extant families (Neopetaliidae, Hemiphlebiidae), and an upshift is associated with Calopterygidae + Polythoridae. When ghost ranges are inferred from the fossil record, many families are shown to have much earlier origination dates. In a phylogenetic context, the number of family-level lineages is shown to be up to twice as high as the fossil record alone suggests through the Cretaceous and Cenozoic, and a logistic increase in richness is detected in contrast to an exponential increase indicated by fossils alone.

**Conclusions:**

Our analysis supports the notion that taxa, which appear to have diversified exponentially using fossil data, may in fact have diversified more logistically. This in turn suggests that one of the major apparent differences between the marine and terrestrial fossil record may simply be an artifact of incomplete sampling. Our results also support previous notions that adult colouration plays an important role in odonate radiation, and that Anisozygoptera should be grouped in a single inclusive taxon with Anisoptera, separate from Zygoptera.

## Background

Understanding past changes in biodiversity is a fundamental part of predicting the future of the Earth's ecosystems [1,2]. Phylogenies and the fossil record provide two complementary windows on temporal variation in biodiversity. The most traditional, palaeontological approach is to document the gain and loss of taxa in the fossil record without recourse to phylogenetic information [3-6]. Although the fossil record provides a direct timescale for observations and contains explicit information about extinction, it is incomplete, and more robust at higher taxonomic levels. A more recent, neontological approach is to analyse the shape of phylogenetic trees of extant taxa, revealing heterogeneity in the net rate of cladogenesis across taxa or through time [7,8]. This allows researchers to compare like with like through identifying sister taxa, but relies on the fossil record to provide a direct timescale, and does not contain explicit information about extinction. Combining fossil with phylogenetic information provides the potential therefore to combine the advantages of both to make more robust inferences about macroevolution [9,10]. In this paper we first summarise phylogenetic information for a long-lived clade of insects, the Odonatoidea (dragonflies and their relatives) and then combine it with fossil information to infer temporal changes in their diversification.

Identifying changes in the rate of diversification is central to understanding macroevolutionary processes. The simplest model of clade growth is an exponential one, where the rate of increase of taxa is constant through time [11,12]. The next simplest alternative is logistic growth, an equilibrium model with the growth of the clade declining as richness rises, through competition. Establishing whether or not clade growth follows expansionist or equilibrial models can therefore contribute towards establishing whether biotic interactions are important in macroevolution, as implied by the Red Queen paradigm [1,13]. Rates of speciation and extinction are also commonly variable across clades [7]. Identifying which evolutionary branches have experienced shifts in their macroevolutionary rates is therefore a first step towards establishing which evolutionary or ecological events might be responsible.

The insects comprise the majority of extant described species. Previous work on insect macroevolution [14] began with the traditional taxic approach from palaeontology [4,15], to which was added the neontological phylogenetic approach [16-19] which has identified a number of evolutionary and ecological processes that have shaped insect diversity [14]. Data have suggested that overall rise in taxonomic richness may be declining modestly towards the present [4], although not strongly so [15], and not in many recent radiations [14]. This is consistent with the generally exponential increase in taxa in the terrestrial fossil record [5,11,12,15] in comparison to the marine record [6]. However the relative incompleteness of the insect record may provide a source of bias, because the true originations of clades likely occurred prior to their first appearance in the fossil record. Using phylogenies to infer ghost ranges, Davis et al. [19] showed that a number of insect orders likely originated prior to their first fossil appearance, making the increase in orders through time look more logistic. The analysis of order-level trends however raises problems; there is a greater risk of paraphyly, complicating the estimation of ghost ranges, and changes in order richness do not necessarily reflect changes at lower taxonomic levels [20]. It would therefore be useful to examine patterns of diversification at a lower taxonomic level, in a clade of insects of equivalent age to the whole class, which previous studies have only achieved using the traditional taxic palaeontological approach.

Despite being less speciose than many other insect orders, the Odonatoidea (Odonata plus the extinct Protodonata) (= Holodonata [21], = Neodonatoptera [22]) are among the most ancient of all living continental fauna, with a fossil record extending back 320 million years, surviving several mass-extinction events [21]. Their accessibility as study systems has informed on many questions in ecology, evolution, and conservation biology [23]. This study aims to summarise existing phylogenetic information on the Odonatoidea by constructing a supertree at the family level. We first apply a neontological approach to the tree to detect where shifts in the net rate of diversification have occurred, and then combine the phylogenetic information with fossil record data to observe patterns of family-lineage richness over time.

## Methods

### Taxonomy

While previous family-level supertree studies of insects [18,24] have used the taxonomic nomenclature of Gordh & Headrick [25] for extant families and Ross & Jarzembowski [26] and the EDNA fossil insect database [http://edna.palass-hosting.org] for extinct families studies, it is evident from examining more recent literature that views of odonate taxonomy have since altered, particularly with respect to fossil families [21]. Using the above references as our starting point, we thoroughly scoured recent literature to build up a taxonomy which represents more recent views, including new families and addressing synonymy. This is provided with references in additional file 1. Any synonyms used in input trees were corrected accordingly to ensure that only one name was used per taxon, preventing the same taxon appearing in the tree in two different positions. The family Eugeropteridae represents the separate order Geroptera and has been used as the supertree outgroup, in line with previous work [19,21,27].

### Input trees

Papers containing Odonata phylogenies were searched for online using Google Scholar, Web of Knowledge and Science Direct databases. References cited in the studies found were used to find additional papers. As input tree searches were carried out until February 2010, no papers published after that time are here considered. Only papers post-1969 were used, being published after the groundbreaking study by Hennig [28] on insect classification and cladistic methodology.

Family-level Odonata phylogenies, constructed from either molecular or morphological data, were collected. In genus- and species-level phylogenies, taxa from the same family were reduced into a single leaf. In the case of a valid family (using our taxonomy) emerging as paraphyletic in a species level tree, all lineages were condensed into a single branch as this has no bearing on relationships with other families, and any valid families that were shown as polyphyletic were removed on grounds of uncertain placement following Davis et al. [18]. Trees where families were grouped together as higher taxa (e.g. into superfamilies) were not used. In total, 32 phylogenies from 23 papers were collected (see additional file 2).

### Supertree methods

Two matrix-based methods were initially used for supertree construction: Standard Matrix Representation with Parsimony (MRP [29,30]), and Matrix Representation with Compatibility (MRC [31]). These methods have been shown to perform well compared to other methods in empirical studies [19] and cope with data sets containing high amounts of conflict [24]. MRP has been shown to perform well in simulation [32], and MRC has been shown to perform similarly well [33]. For each method, a majority rule tree with minority components was constructed from all equally optimal trees returned. Where there is less than a 50% majority for a relationship between particular taxa in equally optimal trees, the relationship appearing more times than any other is taken in the consensus. Fully bifurcating trees were required for diversification analyses (see below). For software and settings see additional file 3. Distance supertree methods were not considered as these have performed poorly compared to matrix-based methods in similar empirical studies [18,19,24]. Methods such as matrix representation with flipping (MRF) and quartet fit (QFIT) were not possible to use as our dataset proved too computationally demanding to run using available software [34,35] without compartmentalising the data set multiple times. Furthermore, we also avoid using strict supertree methods [see 36] which identify only relationships common to all input trees. There is unavoidably conflict between relationships in input trees collected from the literature and the aim of our work is to find an optimum solution rather than only summarise the areas of greatest certainty regarding phylogenetic relationships.

The dataset was then refined to account for data non-independence, following the protocol outlined in Davis et al. [24]. Thirty input trees were used in the final supertree analyses. The refined dataset was then used to construct phylogenies using both the standard MRP and MRC methods.

We use the V index [37] to measure support as used in previous supertree studies [18,19,24,38]. This considers the number of input trees in agreement and in conflict with relationships in the supertree on a scale running from -1 (all conflicting) to +1 (all supporting). An input tree and supertree are in agreement when the relationship in the input tree directly matches that in the supertree (accounting for missing taxa in input trees). Conversely, where there is direct disagreement in a relationship between supertree and input tree, this is conflict. Input trees not relevant to the supertree node in question are not considered. For each supertree node, a score is given (either -1 or +1) for each input tree in the data set. For example, in a supertree study with 10 input trees, the supertree node would receive 10 individual scores which are averaged to give the overall nodal support value. The average score of all nodes gives a total V score for each supertree. Rather than V scores alone, V+ scores were also considered, because as well as relationships found directly in the dataset, they also consider as supporting relationships those that could be permitted by the dataset and do not directly conflict (e.g. polytomies). For a detailed discussion of the V index and supertree support see Wilkinson et al. [37].

### Diversification analysis and taxon age

Significant upshifts and downshifts in the rate of diversification throughout the MRP and MRC majority rule trees were detected using the Slowinski-Guyer measure of imbalance, which compares the species richness of all extant sister clades [39-41]. The number of species in each family was obtained from the World Odonata List [http://www.pugetsound.edu/academics/academic-resources/slater-museum/biodiversity-resources/dragonflies/world-odonata-list]. Any differences between this list and our taxonomy were accounted for (e.g. the inclusion of families we recognise as valid grouped under another family name were separated). Species numbers are shown in Table 1. This measure uses the assumption that, although diversification rates were not constant, at any point in time the diversification between two clades occurred at the same rate. Under this null model, the probability of observing an equal or greater difference in species richness between sister groups is given by 2[N_small_/(N_large_+N_small_-1)]. This calculation is carried out through all extant sister clades in the phylogeny. Assuming that sister groups originated at the same time, effects of the ages of clades on diversification between sister groups can be accounted for. However, with this approach, there is the possibility that significant shifts at nodes will have an effect on those nodes further down the tree, known as the 'trickle-down effect'. The method used by Davies et al. [41] of adjusting the species richness in significant clades was used to eliminate this effect [see [18,19,24,42]]. Alternative methods are available for detecting the location of shifts in diversification across a phylogeny, but are not suitable for our data. For example the SymmeTREE approach [43] employs likelihood-based shift statistics but requires a resolved or nearly-resolved phylogeny at species level. Medusa [44] can be applied to an incompletely-resolved phylogeny but requires a fully dated tree.

**Table 1:** Species richnesses of extant families

Family	Species described	Family	Species described
Aeshnidae	441	Isostictidae	45
Amphipterygidae	12	Lestidae	152
Austropetaliidae	12	Lestoideidae	14
Calopterygidae	176	Libellulidae	986
Chlorocyphidae	151	Lindeniidae	32
Chlorogomphidae	45	Macromiidae	123
Chlorolestidae	35	Megapodagrionidae	193
Coenagrionidae	1121	Neopetaliidae	1
Cordulegastridae	51	Perilestidae	19
Cordulephyidae	5	Petaluridae	12
Corduliidae	244	Platycnemididae	227
Dicteriadidae	2	Platystictidae	214
Epallagidae	69	Polythoridae	59
Epiophlebiidae	2	Protoneuridae	259
Gomphidae	923	Pseudolestidae	1
Hemiphlebiidae	1	Pseudostigmatidae	19
Hypolestidae	116	Synthemistdae	43

Family-level richness over time was also documented. Families which could not be included in the phylogeny, because phylogenetic information is unavailable, were however included when looking at family-level richness over time, to provide as complete a picture as possible. Using literature published up to the end of 2009, the origination dates of known families were taken from their first appearance in the fossil record and extinction dates of extinct taxa from their last appearance. Dates are in line with a recent geological timescale [45,46] (see additional file 4). Using this information, the number of family lineages present in each stage through geological time can be plotted. For a discussion on the use of the term "lineages" here, rather than families *sensu stricto *see Davis et al. [19]. This richness was then corrected according to information in both the MRP and MRC phylogenies, by adjusting family lineage origination dates based on ghost ranges. Assuming that sister taxon lineages must have both been present when the divergence between them occurred, and that differences between the fossil record and phylogenies were caused by an incomplete fossil record and not an incorrect phylogeny, sister lineage origination dates were altered to equal that of the earlier sister group. The effect of both the standard MRP and MRC phylogenies was examined.

### Tests for exponential or logistic increase in family-lineage richness

To conduct time series analysis it is generally preferable to have equally-spaced sampling intervals. This was achieved by generating from the original data, new interpolated time-series of the same length, but equally spaced, using akima splines [47], using the aspline function in the Akima package in R. The expectation from exponential growth is that the logarithm of number of taxa will increase linearly over time. Logistic growth, where the rate of increase in taxon richness declines at high richness, can therefore be inferred from significant non-linearity (i.e. a deceleration of log richness nearer the present), which can be established by comparing the fit of linear and quadratic regressions of log(richness +1) against time. We tested for temporal autocorrelation in the linear regression by the Durbin-Watson test. All residual series showed significant positive autocorrelation at lag 1, and in all cases examination of the autocorrelation and partial autocorrelation functions indicated that the residuals were well described by an autoregression model of order 1 (AR(1)). We therefore accounted for this by using generalized least squares to model the data with an AR(1) residual correlation structure [48], and compared the linear and quadratic models. Modeling was performed in R [49] using the gls function from the nlme library.

## Results

### Phylogeny

**Figure 1:**
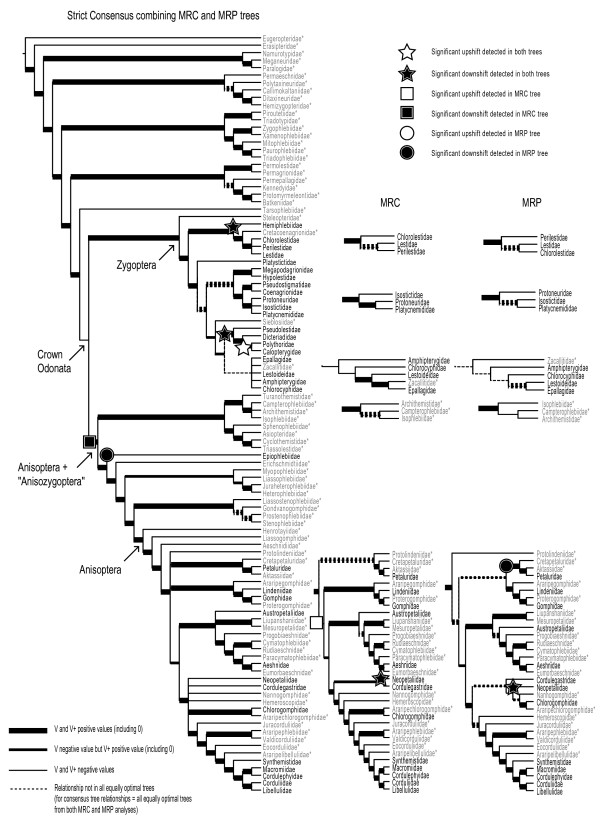
**Supertree phylogeny of Odonatoidea based on MRC and MRP analyses of 30 input trees**. The strict consensus between MRC and MRP supertrees is supplemented where MRC and MRP topologies differ. Taxa in grey marked with * are extinct. Taxa in black are extant.

Both the MRP and the MRC phylogenies (see Figure 1) show the divergence of the Zygoptera (damselflies) and Anisoptera (true dragonflies) + "Anisozygoptera" into two robustly supported monophyletic groups (V scores: Zygoptera, +0.280; Anisoptera + "Anisozygoptera", +0.714). The Anisoptera are nested within a paraphyletic assemblage of "anisozygopteran" families, comprising five main lineages, including the extant family Epiophlebiidae belonging to its own exclusive lineage. The whole crown group of extant odonate families nests within a paraphyletic assemblage of fossil protodonate and odonate families. The backbone of the tree as a whole in both MRP and MRC analyses is robustly supported with primarily positive V scores for clades. The only clade receiving a negative V score is crown group Odonata (V = -0.111), though there is some uncertainty regarding the backbone in Anisoptera, where some clades are not found in all equally optimal trees and there are differences in the most commonly found relationships between MRP and MRC analyses. However, the MRP and MRC trees are largely similar at lower levels (Figure 1). For V and V+ scores of individual clades see additional file 5.

### Diversification

Both the MRP and MRC majority rule supertrees show significant shifts in net rates of diversification (Figure 1). Both show downshifts in the single family lineages Hemiphlebiidae (Zygoptera) and Neopetaliidae (Anisoptera), and a downshift in the lineage leading to Pseudolestidae + Dicteriadidae + Polythoridae + Calopterygidae (Zygoptera), before a subsequent positive shift in the lineage leading to Polythoridae + Calopterygidae. All shifts except that involving Neopetaliidae occur at the base of clades obtaining positive V scores, and in the case of Neopetaliidae, a significant downshift is detected despite differing placement in the two trees. Other upshifts and downshifts detected are shown in just one tree or the other. These shifts are detected in the region of uncertainty regarding the backbone of the Anisoptera clade - a downshift in the Petaluridae lineage (MRP), or a downshift in a clade comprising Epiophlebiidae + Petaluridae + Lindeniidae + Gomphidae + Austropetaliidae + Aeshnidae + Neopetaliidae + Cordulegastridae + Synthemistidae + Macromiidae + Libellulidae + Corduliidae, then a subsequent upshift in this clade to the exclusion of Epiophlebiidae and Petaluridae (MRC). See additional file 6 for sister group species richness comparisons in full.

### Family richness, originations and extinctions

**Figure 2:**
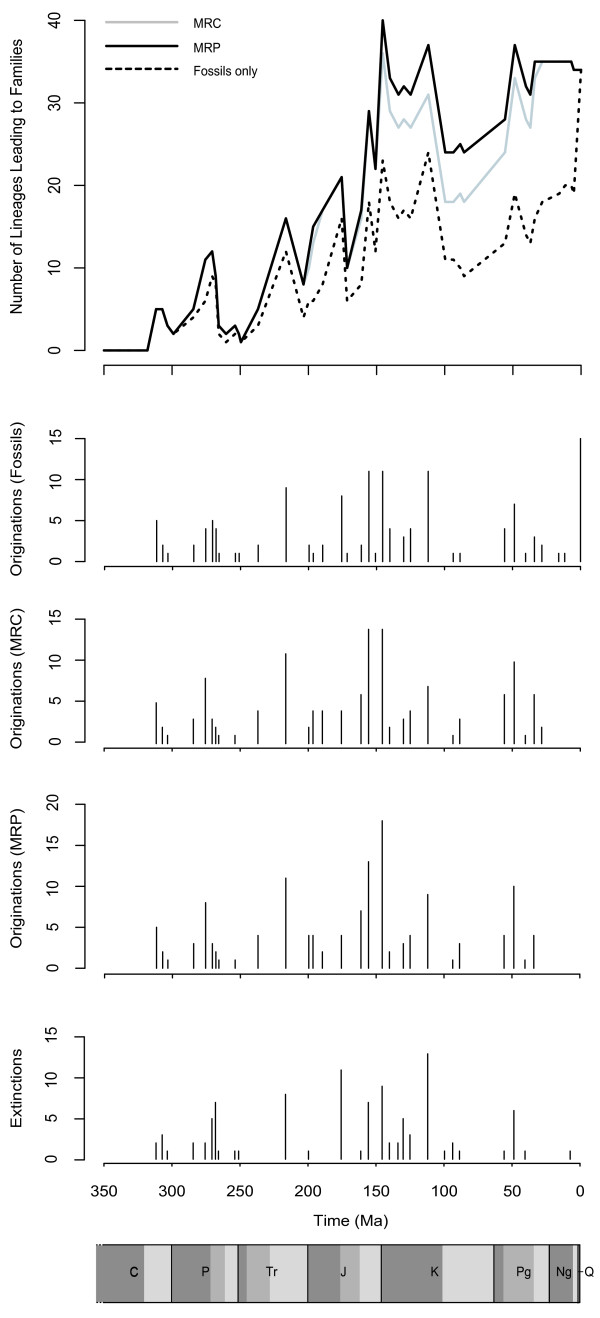
**A lineage through time plot showing family-level richness, origination and extinction events over a geological timescale based on fossil information either alone, or in combination with either MRC or MRP phylogenetic information**. For the geological timescale, C = Carboniferous, P = Permian, Tr = Triassic, J = Jurassic, K = Cretaceous, Pg = Palaeogene, Ng = Neogene, Q = Quaternary. Different coloured bands represent lower, middle (if applicable) and upper stages of each geological period [45,46].

Aside from family extinctions at the end of the Permian and Triassic, a lineage through time plot based on fossil information only (Figure 2) shows an increase in the number of family-level lineages leading up to the Jurassic-Cretaceous boundary. The increase in richness is interrupted by extinctions and origination events throughout the Cretaceous, including a significant extinction at the Lower-Upper Cretaceous boundary, before a sharp rise in richness to the present.

The inclusion of ghost range information shows a slightly different scenario. Both MRP and MRC-based analyses give similar pictures. As expected with ghost ranges inferred, earlier originations occur in the phylogeny-adjusted models, and richness is higher (see Figure 2). With ghost ranges inferred, between 46 (MRC) and 50 (MRP) families have their fossil ranges extended (see additional file 4 for details), with the most dramatic extension for Epiophlebiidae (0 to 196.5 my). Although the same extinction events are apparent, the rise in family-level lineages is slightly sharper in the Triassic and rises dramatically during the Jurassic. The ultimate impact of this is that richness calculated using fossil information alone is only 57-64% of that calculated using phylogenetic information by the end of the Jurassic. Patterns of origination and extinction are similar in all scenarios through the Cretaceous, and while higher richness is evident in the Cenozoic with ghost ranges inferred, the level of richness is stable, compared to the rapid rise in richness found based only on fossil data.

Generalized least squares, accounting for temporal autocorrelation in the residuals, supports the inclusion of a quadratic (i.e. curvilinear) temporal component to log richness for both the MRC supertree (t = -2.65, df = 43, p = 0.011) and the MRP supertree (t = -3.12, df = 43, p = 0.0032). However, for the fossil data alone inclusion of the quadratic term is not well-supported (including extant richness t = -1.49, df = 43, p = 0.144; not including extant richness t = -1.93, df = 42, p = 0.06).

## Discussion

### Odonata phylogeny

Two alternative supertree analyses, representing the largest family-level phylogenetic analysis of Odonatoidea to date, converge on a very similar topology, with overall positive support, indicating general agreement between input trees regarding many relationships within the tree (Figure 1). Therefore based on primary studies from which input trees have been acquired, we can summarise the following consensus regarding relationships within Odonatoidea, following supertree analysis. Importantly, Zygoptera are a monophyletic suborder, separate to Anisoptera (+ "Anisozygoptera"), which refutes previous hypotheses suggesting Zygoptera could be a paraphyletic grade towards Anisoptera [50,51]. "Anisozygoptera", although considered a modern suborder, and regarded as such in recent phylogenetic analyses [e.g. [52]] based on the single extant family Epiophlebiidae, should not be given official taxonomic status. As can be seen, with inclusion of fossil taxa, "Anisozygoptera" is a paraphyletic assemblage with Anisoptera nested within.

It is important that the backbone of the tree receives mostly positive V support, and provides a solid framework to analyse evolutionary questions at a high level in Odonatoidea, though there are some areas of the tree which do remain problematic. The negative support (representing much conflict in the constituent input trees) at the node representing crown Odonata is initially concerning. However, there is much agreement for monophyletic Zygoptera and Anisoptera + "Anisozygoptera" and the negative support for the crown group as a whole represents uncertainty relating to the placement of the fossil family Tarsophlebiidae (i.e. is it included in the crown group or not?). In both supertrees Tarsophlebiidae sits outside the crown group, yet it is represented both within [53,54] and outside [55] of the crown group in input trees, and Bybee et al. [56] show these alternative scenarios in different analyses. Input trees do not concur well over the position of this taxon, and though the debate rolls on, recent fossil evidence supports the more basal position for Tarsophlebiidae that we find here [57].

The most important phylogenetic uncertainty to target in future work must be along the backbone of the Anisoptera clade with most topological differences between the two supertree methods occurring here, and this does affect the result of diversification analyses (see below). However, below the suborder level there is much agreement in relationships. The supertree presented here should be regarded as a work in progress since taxonomic revisions, discovery of new fossil forms, and new molecular data for the order are now commonly reported. Furthermore, the inclusion of more fossil families may help resolve the tree further, but many are yet to be included in phylogenetic analyses.

### Significant shifts in diversification and potential morphological innovations

Previous work by Misof [58] identified two major characteristics within Anisoptera families that are positively correlated with species richness: the evolution of elaborate colour patterns and the evolution of sexual dimorphism in colouration (sexual dimorphism in body size is common in all Anisoptera [59]), features often linked to the process of sexual selection and its role in speciation.

Downshifts in net diversification are inferred from the low extant richness of Hemiphlebiidae, Neopetaliidae (both significant downshifts in both trees), Epiophlebiidae, Petaluridae (both significant downshifts in the MRP tree) and a clade containing Pseudolestidae (significant in both trees, preceding a later upshift in Polythoridae and Calopterygidae). All these families have simple body colouration patterns [60], consistent with Misof [58]. In addition the families Polythoridae and Calopterygidae both show dramatic bright and sexually dimorphic colouration and are associated with a robust diversification upshift in both trees. Our results therefore combine well with Misof's findings by demonstrating where identifiable radiations and restrictions have likely occurred. In addition to these features, Epiophlebiidae also has long larval stages (5-6 y), which could feasibly increase extinction risk or hinder speciation [14]. The Neopetaliidae has extremely specialised niches (riverside caves) possibly restricting its diversification. In the latter two cases though, such characteristics might just be those of surviving species rather than the families as a whole, so caution is required. A fuller determination of the causes of any shifts would benefit from an analysis at finer taxonomic resolutions. The use of simple topology-based statistics in the absence of fossil data and extinct taxa likely limits the inferences that can be made on diversification shifts, and may induce biases [see [61,62]]. Hence the findings presented here should be revisited as the opportunity to incorporate new data and more complex, and potentially realistic, diversification models presents itself.

### Family richness through time

Fossil record-only analysis follows some similar short term variation to the phylogeny-based analyses, but phylogeny-based analyses show some noticeably higher increases in family-level lineage richness through the Triassic and Jurassic, and we show that richness was likely much higher through the Cretaceous and then consequently the Cenozoic than can be shown by using the fossil record alone (Figure 2). It is most probable that the extensions of fossil ranges we infer are quite conservative [63], but the impact of including such information is evident in our analyses. The divergence between the fossil and phylogenetic richness curves (Figure 2) accumulates gradually throughout the timeline, and is notable towards the end of the Jurassic, suggesting that many lineages with much later first fossils are rather first found here. This may of course partly represent an abundance of suitable fossil-bearing strata instead of, or as well as, a genuine set of first originations. It therefore remains possible that the taxa involved actually have still earlier origination dates. If true however, this would only strengthen our findings by making the true richness curve appear even more logistic. Using fossil data alone there is a suggestion of a slow-down in the net rate of origination that is not significant, mirroring the insect fossil record as a whole [4]. Both the phylogenetic analyses however alter the fossil-based curve to a more logistic model, with a sudden increase in family richness through the Jurassic being the most noticeable difference. There has been debate in the past over whether the fossil record for all organisms exhibits logistic growth, or whether it has been exponential but punctuated with mass extinctions that limit the level of taxonomic diversity [64]. However, given that an approximately exponential increase in richness can turn to a logistic one after accounting for gaps in the fossil record, our results suggest that caution should be applied to other such cases, particularly in the terrestrial record where the evidence for logistic growth has so far been thinner [11,12,15].

After accounting for the general trend in taxonomic richness, variation around this trend remains. Some of this coincides with well-documented mass-extinctions and recoveries, including here the end-Permian, but in other cases, such as the end-Cretaceous, no effect is seen [see also 65]. In the fossil record as a whole, fluctuations are known to correlate with a number of environmental and geophysical variables [66,67]. The sudden increase in richness in the Jurassic coincides with an icehouse climate mode [68], as is the case for the initial radiation of Odonatoidea in the Carboniferous, consistent with the findings of Mayhew et al. [66]. Also, notable changes to freshwater ecosystems occurred in the Jurassic with the radiation of aquatic Diptera and other animal taxa that are today important food sources for Odonata [69]. Though the environmental correlates of Odonata richness through time requires a fuller study, our results suggest that such studies could benefit from incorporation of phylogenetic information.

## Conclusions

Phylogenetic information provides one way to adjust for the imperfections and biases inherent in analyses of fossil data. When such information is used to address family-level richness in an ancient clade of insects, different models of macroevolution are supported. The more logistic pattern of diversification we observe when phylogenetic information is included in such a study is in contrast to the exponential pattern of increases in odonate richness suggested by the fossil record alone, and the terrestrial record more generally. These results therefore imply that some of the major differences between the marine and terrestrial records are artifacts of incomplete sampling. Our supertrees provide a baseline for further macroevolutionary, comparative and phylogenetic studies of Odonata and Protodonata.

## Authors' contributions

All authors contributed to manuscript writing and have read and approved the final manuscript. RBD and ELRS collected input trees and conducted supertree analyses. RBD and DBN drew up a valid taxonomy and DBN collected all the information regarding origination and extinctions of lineages. RBD conducted sister group species richness analyses and constructed the lineage through time plots. PJM tested for logistic/exponential clade growth and coordinated the study as a whole.

## Supplementary Material

Additional file 1 **Valid taxonomy**. A list of Odonata and Protodonata family names which are considered valid for the purposes of this study. Click here for file

Additional file 2 **Input trees**. Input trees from primary literature used in supertree analysis. Click here for file

Additional file 3 **Software & settings**. Software used and settings applied for both MRP and MRC supertree analysis. Click here for file

Additional file 4 **Origination & extinction dates**. Fossil first appearance dates and extinction dates for all Odonata and Protodonata families, and modified origination dates when ghost ranges are taken into account. Click here for file

Additional file 5 **V and V+ scores**. V and V+ support scores for MRP and MRC supertree nodes. Click here for file

Additional file 6 **Sister group species richness comparisons**. Sister group species richness comparisons for both MRP and MRC supertrees indicating where significant upshifts and downshifts in species richness have occurred. Click here for file
