# Supplementary material for: Fossil gaps inferred from phylogenies alter the apparent nature of diversification in dragonflies and their relatives
Source: BMC Evol Biol. 2011 Sep 14;11:252. doi: 10.1186/1471-2148-11-252 (PMC3179963; doi:10.1186/1471-2148-11-252)
Supplement: Additional file 1 — Valid taxonomy. A list of Odonata and Protodonata family names which are considered valid for the purposes of this study. [file 1471-2148-11-252-S1.PDF]

ADDITIONAL FILE 1 – VALID TAXONOMY

Below is what we consider to be a valid family-level taxonomy of odonate and protodonate families. Extant families are from Gordh & Headrick (2000). Most extinct families are mentioned in Ross & Jarzambowski (1993) or recorded at a later date in the EDNA fossil insect database. For families missing from both Ross & Jarzembowski (1993) and EDNA, references are provided which establish or confirm their (re)erection. Common synonyms are given in addition. Those indicated with * are extinct taxa.

Note that several of the generic and familial assignments used in EDNA are as they were at the time of the original descriptions and have often not been updated following the significant revisions in recent years. This can lead to a family being absent from the EDNA database while the genera and species comprising the fossil record for that family are not. Note also that the palaeoentomological community commonly considers as families many taxa which are considered by neontologists to belong within other families (e.g. Alloptetaliidae, Gomphaeschnidae, Idionychidae and Dysagrionidae), subsume some families into others which neontologists usually consider separate (e.g. Synthemistidae) or simply prefer one synonym over another (e.g. Selenothemistidae vs. Turanothemistidae, Chlorolestidae vs. Synlestidae).

We recognise that the definitions and relationships of extant odonate families has been in a state of flux in recent years and are becoming increasing elucidated by molecular, rather than morphological, characters (see references for input trees). This is in contrast to fossil taxonomy, based mostly on characters in wing impressions, which could be considered as less well developed and difficult to integrate with modern, extant systematics. What we provide here, we hope, is a reasonable summary of current views on valid family-level taxonomy from which to perform our analyses.

Aeschnidiidae*	
Aeshnidae = Allopetaliidae, Gomphaeschnidae	
Aktassiidae*	
Amphipterygidae	
Araripechlorogomphidae*	
Araripegomphidae*	
Araripelibellulidae*	
Araripephlebiidae*	
Archithemistidae*	
Asiopteridae* = Oreopteridae	
Austroperilestidae*	
Austropetaliidae	
Batkeniidae*	
Bechlyidae*	
Bolcacorduliidae*	
Bolcathoridae*	
Callimokaltaniidae*	Huguet et al. (2002)
Calopterygidae = Agriidae	
Campterophlebiidae* = Karatawiidae	
Camptotaxineuridae*	Huguet et al. (2002)
Campylopteridae*	
Chlorocyphidae = Dicteriastidae	
Chlorogomphidae	
Chlorolestidae = Synlestidae, Chorismagrionidae	
Coenagrionidae = Agrionidae, Coenagriidae	
Cordulegastridae	
Cordulephyidae	
Corduliidae = Idionychidae	
Cretacoenagrionidae*	Jarzembowski et al. (1998)
Cretapetaluridae*	
Cyclothemistidae*	
Cymatophlebiidae*	
Dicteriadidae = Heliocharitidae	

Ditaxineuridae*	
Enigmaeshnidae*	
Eocorduliidae*	Bechly (1996)
Eosagrionidae*	
Epallagidae = Euphaeidae	
Epiophlebiidae	
Erasipteridae*	
Erichschmidtiidae*	Bechly (1996)
Eumorbaeschnidae*	
Eugeropteridae*	
Euthemistidae*	
Frenguelliidae*	
Gomphidae	
Gondvanogomphidae*	Bechly (1996); Schlüter (2003)
Hemeroscopidae*	
Hemiphlebiidae	
Hemizygopteridae*	Huguet et al. (2002)
Henrotayiidae*	
Heterophlebiidae*	
Hypolestidae	
Isophlebiidae*	
Isostictidae	
Juracorduliidae*	Bechly & Ueda, (2002)
Juragomphidae*	
Juraheterophlebiidae*	
Juralibellulidae*	
Kaltanoneuridae*	
Kargalotypidae*	Nel et al. (2001)
Kennedyidae*	
Kohlwaldiidae*	Zessin (2008)
Lapeyriidae*	Nel et al. (1999)
Latibasaliidae*	
Lestidae	
Lestoideidae = Diphlebiidae, Philogangidae	
Liadotypidae*	Nel et al. (2001)
Liassogomphidae* = Gomphitidae	
Liassophlebiidae*	
Liassostenophlebiidae*	
Libellulidae	
Lindeniidae	
Liupanshaniidae*	
Macromiidae	
Meganeuridae* = Tupidae	
Megapodagrionidae = Dysagrionidae, Congqingiidae, Euarchistigmatidae, Thaumatoneuridae	
Mesochlorogomphidae*	
Mesomantidiidae*	
Mesuropetalidae*	
Mitophlebiidae*	
Myopophlebiidae*	Fleck et al. (2003)
Namurotypidae*	Bechly (1996); Zessin (2006)
Nannogomphidae*	
Neopetaliidae	
Nodalulaidae *	
Nothomacromiidae* = Pseudomacromiidae	
Oboraneuridae*	Zessin (2008)
Palaeomacromiidae* = Bolcathemidae	
Paracymatophlebiidae*	

Paragonophlebiidae*	
Parastenophlebiidae*	
Paralogidae*	
Paurophlebiidae*	Vasilenko & Rasnitsyn (2007)
Perilestidae	
Permaeschnidae*	
Permagrionidae* = Permagriidae	
Permepallagidae*	
Permolestidae*	
Permophlebiidae*	
Petaluridae	
Pholidoptilidae*	Huguet et al. (2002)
Piroutetiidae*	Nel et al. (2001)
Platycnemididae	
Platystictidae	
Polythoridae = Polyphoridae	
Polytaxineuridae*	
Priscalestidae*	
Progobiaeshnidae*	
Prohemeroscopidae*	
Prostenophlebiidae*	Fleck et al. (2003)
Proterogomphidae*	
Protolindeniidae*	
Protomyrmeleontidae* = Triassagrionidae	
Protoneuridae	
Pseudolestidae	
Pseudostigmatidae	
Rudiaeschnidae*	Bechly et al. (2001)
Saxonagrionidae*	
Sieblosiidae*	
Sonidae*	
Sphenophlebiidae*	Bechly (1997); Fleck et al. (2004)
Steleopteridae*	
Stenophlebiidae*	
Synthemistidae = Synthemidae	
Tarsophlebiidae*	
Triadophlebiidae*	
Triadotypidae* = Reisiidae	
Triassolestidae* = Italophlebiidae, Mesophlebiidae, Progonophlebiidae, Triassoneuridae, Triassothemidae	
Turanothemistidae* = Selenothemistidae	
Valdicorduliidae*	Bechly (1996)
Xamenophlebiidae*	
Zacallitidae*	
Zygophlebiidae*	

References

Bechly, G. 1996 Morphologische Untersuchungen am Flügelgeäder der rezenten Libellen und deren Stammgruppenvertreter (Insecta: Pterygota: Odonata), unter besonderer Berücksichtigung der Phylogenetischen Systematik und des Grundplanes der Odonata . *Petalura*, **Special Volume 2**, 1–402.

Bechly, G. 1997 New fossil odonates from the Upper Triassic of Italy, with a redescription of *Italophlebia gervasuttii* Whalley, and a reclassification of Triassic dragonflies (Insecta: Odonata). *Rivista del Museo Civico di Scienze Naturali "Enrico Caffi"*, **19**, 31–70.

Bechly, G., Nel, A., Martínez-Delclòs, X., Jarzembowski, E. A., Coram, R., Martill, D., Fleck, G., Escuillié,

F., Wisshak, M. M. & Maisch, M. 2001 A revision and phylogenetic study of Mesozoic Aeshnoptera, with description of numerous new taxa (Insecta: Odonata: Anisoptera). *Neue Paläontologische Abhandlungen*, **4**, 1–219.

Bechly, G. & Ueda, K. 2002 The first fossil record and first New World record for the dragonfly clade Chlorogomphida (Insecta: Odonata: Anisoptera: Araripechlorogomphidae n. fam.) from the Crato Limestone (Lower Cretaceous, Brazil). *Stuttgarter Beiträge zur Naturkunde Serie B (Geologie und Paläontologie)*, **328**, 1–11.

Fleck, G., Bechly, G., Martínez-Delclòs, X., Jarzembowski, E., Coram, R. & Nel, A. 2003 Phylogeny and classification of the Stenophlebioptera (Odonata: Epiproctophora). *Annales de la Société entomologique de France (Nouvelle série)*, **39**, 55–93.

Fleck, G., Bechly, G., Martínez-Delclòs, X., Jarzembowski, E. A. & Nel, A. 2004 A revision of the Upper Jurassic-Lower Cretaceous dragonfly family Tarsophlebiidae, with a discussion on the phylogenetic positions of the Tarsophlebiidae and Sieblosiidae (Insecta, Odonatoptera, Panodonata). *Geodiversitas*, **26**, 33–60.

Gordh, G. & Headrick, D. H. 2000 A Dictionary of Entomology. Wallingford: CABI Publishing.

Jarzembowski, E. A., Martínez-Delclòs, X., Bechly, G., Nel, A., Coram, R. & Escuillié, F. 1998 The Mesozoic non-calopterygoid Zygoptera: description of new genera and species from the Lower Cretaceous of England and Brazil and their phylogenetic significance (Odonata, Zygoptera, Coenagrionoidea, Hemiphlebioidea, Lestoidea). *Cretaceous Research*, **19**, 403–444.

Huguet, A., Nel, A., Martínez-Delclòs, X., Bechly, G. & Martins-Neto, R. 2002 Preliminary phylogenetic analysis of the Protanisoptera (Insecta: Odonatoptera). *Geobios*, **35**, 537–560.

Nel, A., Béthoux, O., Bechly, G., Martínez-Delclòs, X., and Papier, F. 2001 The Permo-Triassic Odonatoptera of the “Protodonate” grade (Insecta: Odonatoptera). *Annales de la Société entomologique de France (Nouvelle série)*, **37**, 501–525.

Nel, A., Gand, G., & Garric, J. 1999 A new family of Odonatoptera from the continental Upper Permian: The Lapeyriidae (Lodève Basin, France). *Geobios*, **32**, 63–72.

Ross, A. J. & Jarzembowski, E. A. 1993 Arthropoda (Hexapoda: Insecta). In *The Fossil Record 2* (ed M. J. Benton) pp. 363–426. London: Chapman and Hall.

Schlüter, T. 2003 Fossil insects in Gondwana - localities and palaeodiversity trends. *Acta zoologica cracoviensia*, **46** (suppl.- Fossil Insects), 345–371.

Vasilenko, D. V. & Rasnitsyn, A. P. (2007) Fossil ovipositions of dragonflies: review and interpretation. *Paleontological Journal*, **41**, 1156–1161.

Zessin, W. 2006 Zwei neue Insektenreste (Megasecoptera, Odonatoptera) aus dem Westfalium D (Oberkarbon) des Piesberges bei Osnabrück, Deutschland. *Virgo, Mitteilungsblatt des Entomologischen Vereins Mecklenburg*, **9**, 37–45.

Zessin, W. 2008 Überblick über die paläozoischen Libellen (Insecta, Odonatoptera). *Virgo, Mitteilungsblatt des Entomologischen Vereins Mecklenburg*, **11**, 5–32.
