# Supplementary material for: Fossil gaps inferred from phylogenies alter the apparent nature of diversification in dragonflies and their relatives
Source: BMC Evol Biol. 2011 Sep 14;11:252. doi: 10.1186/1471-2148-11-252 (PMC3179963; doi:10.1186/1471-2148-11-252)
Supplement: Additional file 2 — Input trees. Input trees from primary literature used in supertree analysis. [file 1471-2148-11-252-S2.PDF]

ADDITIONAL FILE 2 – INPUT TREES

Journal	Volume	Start Page	Figure	Data	Status in Final Analysis	Comments
Petalura	2	1	-	Morphology	Half	Constructed "bye eye" but contains fossil taxa not in other studies
Cladistics	23	1	2	Morphology, 12S, 16S, 18S, 28S, COII, H3	Half	
Cladistics	23	1	3	Morphology, 12S, 16S, 18S, 28S, COII, H3	Half	
Zootaxa	87	1	1	Morphology	Full	
Societas Internationalis Odonatologica Rapid Communications	4	1		Morphology	Half	Constructed "bye eye" but contains fossil taxa not in other studies
Arthropod Systematics & Phylogeny	66	3	1	12S, 16S, EF1a	Full	
Systematic Entomology	35	6	2	5.8S, 18S, ITS1, ITS2	Full	
Annales de la Société Entomologique de France	39	5	40	Morphology	Full	
Geodiversitas	26	33	9	Morphology	Removed	More recent but far less comprehensive than Rehn (2003)
Journal of Zoological Systematics & Evolutionary Research	46	310	2	12S, 16s, Valine	Half	
Journal of Zoological Systematics & Evolutionary Research	46	310	3	12S, 16s, Valine	Half	
Proceedings of the Royal Society of London B	266	525	9	Morphology	Full	
Molecular Phylogenetics & Evolution	42	339	1 (left)	16S	Full	
Molecular Phylogenetics & Evolution	42	339	1 (right)	EF1a	Full	
Molecular Phylogenetics & Evolution	42	339	2	16S, EF1a, ND1	Full	
Entomological Science	9	55	3a	28S	Half	
Entomological Science	9	55	3b	28S	Half	
Cladistics	18	313	3	18S	Full	
Cladistics	18	313	4	28S	Removed	Covered by Hasegawa & Kasuya (2006)
Cladistics	18	313	5	18S, 28S	Full	
Cretaceous Research	17	67	12	Morphology	Full	
Cretaceous Research	23	439	3	Morphology	Full	
Molecular Biology & Evolution	18	27	5	12S, 16S	Full	
Cladistics	19	432	2	18S, 28S, H3	Full	
Systematic Entomology	33	511	6 (all trees same)	Morphology	Full	
International Journal of Odonatology	8	259	26	Morphology	Full	
Systematic Entomology	28	181	4 (same as 6)	Morphology	Full	
Annals of the Entomological Society of America	96	693	1	12S	Half	
Annals of the Entomological Society of America	96	693	2	12S	Half	
Tree shown in Rehn (2003)			7	Morphology	Half	Using the tree shown in Rehn (2003), but contains fossil taxa not in other studies
Molecular Phylogenetics & Evolution	45	289	2	16S, 28S	Half	
Molecular Phylogenetics & Evolution	45	289	3	16S, 28S	Half	
