# Supplementary material for: Fossil gaps inferred from phylogenies alter the apparent nature of diversification in dragonflies and their relatives
Source: BMC Evol Biol. 2011 Sep 14;11:252. doi: 10.1186/1471-2148-11-252 (PMC3179963; doi:10.1186/1471-2148-11-252)
Supplement: Additional file 3 — Software & settings. Software used and settings applied for both MRP and MRC supertree analysis. [file 1471-2148-11-252-S3.PDF]

ADDITIONAL FILE 3 – SOFTWARE & SETTINGS

The Odonatoidea data set is computationally intensive to run and the whole data set could not be analysed as one using the most thorough search criteria. Therefore, an initial MRC analysis using the computationally least demanding search option was run initially 1000 times to obtain a rough initial estimate of the phylogeny. This was subjected to analysis of robustness using the V index (Wilkinson et al 2005) and the analysis was compartmentalised into two roughly equally sized data sets by splitting the tree recovered in this first analysis at the node which obtained the highest level of support along the backbone of the tree (the highest support for any node is +0.840 but includes just 10 taxa and does not sufficiently well “half” the data set). This node represents Anisoptera + Anisozygoptera (excluding Turanothemistidae, Archithemistidae, Isophlebiidae, Campterophlebiidae, Sphenophlebiidae, Asiopteridae, Cyclothemistidae and Triassolestidae) (V = +0.727). When running one half of the analysis, representative taxa (i.e. those with highest representation as “0”s and “1s” in the matrix) from the other half were included to combat any issues regarding paraphyly, and to check if running smaller analyses would impact adversely on broad relationships between a compartmentalised and full analysis. They do not.

MRC: Using program PARS from the PHYLIP package (Felsenstein 2005). Default options used except number of trees to save (option V) increased to 1000000, input order jumbled (J), and run 1000 analysis run 1000 times, threshold set to 2.0 (as required for compatibility analysis) and Eugeropteridae set as outgroup.

MRP: Using PAUP* (Swofford 2003), Eugeropteridae set as outgroup, heuristic search using tree bisection-reconnection (TBR), random input order of taxa, and 1000 replicates.

Software References:

Felsenstein, J. 2005 PHYLIP (Phylogeny Inference Package) version 3.6. *Distributed by the author. Department of Genome Sciences, University of Washington, Seattle.*

Swofford, D. L. 2003 PAUP*. Phylogenetic Analysis Using Parsimony (*and Other Methods). Version 4. Sinauer Associates, Sunderland, Massachusetts.
