# Supplementary material for: Fossil gaps inferred from phylogenies alter the apparent nature of diversification in dragonflies and their relatives
Source: BMC Evol Biol. 2011 Sep 14;11:252. doi: 10.1186/1471-2148-11-252 (PMC3179963; doi:10.1186/1471-2148-11-252)
Supplement: Additional file 4 — Origination & extinction dates. Fossil first appearance dates and extinction dates for all Odonata and Protodonata families, and modified origination dates when ghost ranges are taken into account. [file 1471-2148-11-252-S4.PDF]

ADDITIONAL FILE 4 – ORIGINATION & EXTINCTION DATES

Provided below is information on all family-level taxa considered valid in this study. Dates are given for first fossil appearances, final fossil appearances (i.e. extinction dates) for extinct taxa (which are indicated by *), first absence dates (i.e. the stratum immediately after final fossil appearances), and phylogenetically-adjusted dates of origin based on both he MRC and MRP phylogeny. Those taxa in grey lack phylogenetic information though dates are given under the phylogenetically-adjusted date columns as they are included in lineage through time plots.

Family	Earliest Stratum	Minimum First Appearance Date	Latest Stratum	Minimum Final Appearance Date	Earliest Stratum Absent	Minimum First Absence Date	Minimum First Appearance Date (MRC)	Minimum First Appearance Date (MRP)
Aeschnidiidae*	Kimmeridgian (J)	150.8	Cenomanian (K)	93.6	Turonian (K)	88.6	161.2	161.2
Aeshnidae = Allopetaliidae, Gomphaeschnidae	Tithonian (J)	145.5					145.5	145.5
Aktassiidae*	Oxfordian (J)	155.6	Barremian (K)	125.0	Aptian (K)	112.0	155.6	155.6
Amphipterygidae		0.0					48.6	48.6
Araripechlorogomphidae*	Aptian (K)	112.0	Aptian (K)	112.0	Albian (K)	99.6	112.0	112.0
Araripegomphidae*	Aptian (K)	112.0	Aptian (K)	112.0	Albian (K)	99.6	145.5	145.5
Araripelibellulidae*	Berriasian (K)	140.2	Aptian (K)	112.0	Albian (K)	99.6	140.2	140.2
Araripephlebiidae*	Aptian (K)	112.0	Aptian (K)	112.0	Albian (K)	99.6	130.0	130.0
Archithemistidae*	Rhaetian (Tr)	199.6	Toarcian (J)	175.6	Aalenian (J)	171.6	199.6	199.6
Asiopteridae* = Oreopteridae	Toarcian (J)	175.6	Oxfordian (J)	155.6	Kimmeridgian (J)	150.8	216.5	216.5
Austroperilestidae*	Ypresian (Pg)	48.6	Ypresian (Pg)	48.6	Lutetian (Pg)	40.4	48.6	48.6
Austropetaliidae		0.0					161.2	161.2
Batkeniidae*	Anisian (Tr)	237.0	Carnian (Tr)	216.5	Norian (Tr)	203.6	237.0	237.0
Bechlyidae*	Moscovian (C)	307.2	Moscovian (C)	307.2	Kasimovian (C)	303.4	307.2	307.2
Bolcacorduliidae*	Ypresian (Pg)	48.6	Ypresian (Pg)	48.6	Lutetian (Pg)	40.4	48.6	48.6
Bolcathoridae*	Ypresian (Pg)	48.6	Ypresian (Pg)	48.6	Lutetian (Pg)	40.4	48.6	48.6
Callimokaltaniidae*	Roadian (P)	268.0	Roadian (P)	268.0	Wordian (P)	265.8	275.6	275.6
Calopterygidae = Agriidae	Priabonian (Pg)	33.9					33.9	33.9
Campterophlebiidae* = Karatawiidae	Sinemurian (J)	189.6	Berriasian (K)	140.2	Valanginian (K)	133.9	189.6	199.6
Camptotaxineuridae*	Artinskian (P)	275.6	Artinskian (P)	275.6	Kungurian (P)	270.6	275.6	275.6
Campylopteridae*	Kasimovian (C)	303.4	Kasimovian (C)	303.4	Gzhelian (C)	299.0	303.4	303.4
Chlorocyphidae = Dicteriastidae		0.0					48.6	48.6
Chlorogomphidae		0.0					112.0	112.0
Chlorolestidae = Synlestidae, Chorismagrionidae	Tithonian (J)	145.5					145.5	145.5
Coenagrionidae = Agrionidae, Coenagriidae	Aptian (K)	112.0					112.0	112.0
Cordulegastridae	Rupelian (Pg)	28.4					28.4	145.5
Cordulephyidae	Thanetian (Pg)	55.8					55.8	55.8
Corduliidae = Idionychidae	Ypresian (Pg)	48.6					88.6	88.6
Cretacoenagrionidae*	Hauterivian (K)	130.0	Hauterivian (K)	130.0	Barremian (K)	125.0	145.5	145.5
Cretapetaluridae*	Berriasian (K)	140.2	Aptian (K)	112.0	Albian (K)	99.6	155.6	155.6
Cyclothemistidae*	Carnian (Tr)	216.5	Toarcian (J)	175.6	Aalenian (J)	171.6	216.5	216.5
Cymatophlebiidae*	Callovian (J)	161.2	Barremian (K)	125.0	Aptian (K)	112.0	161.2	161.2
Dicteriadidae = Heliocharitidae		0.0					33.9	33.9
Ditaxineuridae*	Artinskian (P)	275.6	Kungurian (P)	270.6	Roadian (P)	268.0	275.6	275.6
Enigmaeshnidae*	Cenomanian (K)	93.6	Cenomanian (K)	93.6	Turonian (K)	88.6	93.6	93.6
Eocorduliidae*	Berriasian (K)	140.2	Berriasian (K)	140.2	Valanginian (K)	133.9	140.2	140.2
Eosagrionidae*	Toarcian (J)	175.6	Toarcian (J)	175.6	Aalenian (J)	171.6	175.6	175.6
Epallagidae = Euphaeidae	Ypresian (Pg)	48.6					48.6	48.6
Epiophlebiidae		0.0					196.5	196.5
Erasipteridae*	Bashkirian (C)	311.7	Moscovian (C)	307.2	Kasimovian (C)	303.4	311.7	311.7
Erichschmidtiidae*	Oxfordian (J)	155.6	Oxfordian (J)	155.6	Kimmeridgian (J)	150.8	196.5	196.5
Eumorbaeschnidae*	Tithonian (J)	145.5	Tithonian (J)	145.5	Berriasian (K)	140.2	145.5	145.5
Eugeropteridae*	Bashkirian (C)	311.7	Bashkirian (C)	311.7	Moscovian (C)	307.2	311.7	311.7
Euthemistidae*	Oxfordian (J)	155.6	Oxfordian (J)	155.6	Kimmeridgian (J)	150.8	155.6	155.6
Frenguelliidae*	Ypresian (Pg)	48.6	Ypresian (Pg)	48.6	Lutetian (Pg)	40.4	48.6	48.6
Gomphidae		0.0					145.5	145.5
Gondvanogomphidae*	Aptian (K)	112.0	Aptian (K)	112.0	Albian (K)	99.6	155.6	155.6
Hemeroscopidae*	Aptian (K)	112.0	Albian (K)	99.6	Cenomanian (K)	93.6	145.5	145.5
Hemiphlebiidae	Tithonian (J)	145.5					145.5	145.5
Hemizygopteridae*	Kungurian (P)	270.6	Kungurian (P)	270.6	Roadian (P)	268.0	275.6	275.6
Henrotayiidae*	Toarcian (J)	175.6	Toarcian (J)	175.6	Aalenian (J)	171.6	175.6	175.6
Heterophlebiidae*	Sinemurian (J)	189.6	Toarcian (J)	175.6	Aalenian (J)	171.6	189.6	189.6
Hypolestidae	Priabonian (Pg)	33.9					125.0	125.0
Isophlebiidae*	Aalenian (J)	171.6	Valanginian (K)	133.9	Hauterivian (K)	130.0	189.6	199.6
Isostictidae	Aptian (K)	112.0					112.0	112.0
Juracorduliidae*	Tithonian (J)	145.5	Tithonian (J)	145.5	Berriasian (K)	140.2	145.5	145.5
Juragomphidae*	Oxfordian (J)	155.6	Oxfordian (J)	155.6	Kimmeridgian (J)	150.8	155.6	155.6
Juraheterophlebiidae*	Oxfordian (J)	155.6	Oxfordian (J)	155.6	Kimmeridgian (J)	150.8	189.6	189.6
Juralibellulidae*	Callovian (J)	161.2	Callovian (J)	161.2	Oxfordian (J)	155.6	161.2	161.2
Kaltanoneuridae*	Roadian (P)	268.0	Roadian (P)	268.0	Wordian (P)	265.8	268.0	268.0
Kargalotypidae*	Wordian (P)	265.8	Wordian (P)	265.8	Capitanian (P)	260.4	265.8	265.8
Kennedyidae*	Artinskian (P)	275.6	Carnian (Tr)	216.5	Norian (Tr)	203.6	275.6	275.6
Kohlwaldiidae*	Moscovian (C)	307.2	Moscovian (C)	307.2	Kasimovian (C)	303.4	307.2	307.2

Lapeyriidae*	Kungurian (P)	270.6	Kungurian (P)	270.6	Roadian (P)	268.0	270.6	270.6
Latibasaliidae*	Thanetian (Pg)	55.8	Thanetian (Pg)	55.8	Ypresian (Pg)	48.6	55.8	55.8
Lestidae	Thanetian (Pg)	55.8					55.8	145.5
Lestoideidae = Diphlebiidae, Philogangidae		0.0					48.6	48.6
Liadotypidae*	Kungurian (P)	270.6	Kungurian (P)	270.6	Roadian (P)	268.0	270.6	270.6
Liassogomphidae* = Gomphitidae	Toarcian (J)	175.6	Toarcian (J)	175.6	Aalenian (J)	171.6	175.6	175.6
Liassophlebiidae*	Hettangian (J)	196.5	Toarcian (J)	175.6	Aalenian (J)	171.6	196.5	196.5
Liassostenophlebiidae*	Toarcian (J)	175.6	Toarcian (J)	175.6	Aalenian (J)	171.6	175.6	175.6
Libellulidae	Turonian (K)	88.6					88.6	88.6
Lindeniidae	Aptian (K)	112.0					145.5	145.5
Liupanshaniidae*	Barremian (K)	125.0	Turonian (K)	88.6	Coniacian (K)	85.8	155.6	155.6
Macromiidae	Burdigalian (Ng)	16.0					55.8	55.8
Meganeuridae* = Tupidae	Bashkirian (C)	311.7	Roadian (P)	268.0	Wordian (P)	265.8	311.7	311.7
Megapodagrionidae = Dysagrionidae, Congqingiidae, Eu	Barremian (K)	125.0					125.0	125.0
Mesochlorogomphidae*	Barremian (K)	125.0	Barremian (K)	125.0	Aptian (K)	112.0	125.0	125.0
Mesomantidiidae*	Carnian (Tr)	216.5	Carnian (Tr)	216.5	Norian (Tr)	203.6	216.5	216.5
Mesuropetalidae*	Oxfordian (J)	155.6	Valanginian (K)	133.9	Hauterivian (K)	130.0	155.6	155.6
Mitophlebiidae*	Carnian (Tr)	216.5	Carnian (Tr)	216.5	Norian (Tr)	203.6	216.5	216.5
Myopophlebiidae*	Toarcian (J)	175.6	Toarcian (J)	175.6	Aalenian (J)	171.6	196.5	196.5
Namurotypidae*	Bashkirian (C)	311.7	Bashkirian (C)	311.7	Moscovian (C)	307.2	311.7	311.7
Nannogomphidae*	Tithonian (J)	145.5	Tithonian (J)	145.5	Berriasian (K)	140.2	145.5	145.5
Neopetaliidae		0.0					28.4	145.5
Nodalulaidae*	Aptian (K)	112.0	Aptian (K)	112.0	Albian (K)	99.6	112.0	112.0
Nothomacromiidae* = Pseudomacromiidae	Aptian (K)	112.0	Aptian (K)	112.0	Albian (K)	99.6	112.0	112.0
Oboraneuridae*	Sakmarian (P)	284.4	Sakmarian (P)	284.4	Artinskian (P)	275.6	284.4	284.4
Palaeomacromiidae* = Bolcathemidae	Thanetian (Pg)	55.8	Ypresian (Pg)	48.6	Lutetian (Pg)	40.4	55.8	55.8
Paracymatophlebiidae*	Oxfordian (J)	155.6	Oxfordian (J)	155.6	Kimmeridgian (J)	150.8	155.6	155.6
Paragonophlebiidae*	Oxfordian (J)	155.6	Tithonian (J)	145.5	Berriasian (K)	140.2	155.6	155.6
Parastenophlebiidae*	Tithonian (J)	145.5	Tithonian (J)	145.5	Berriasian (K)	140.2	145.5	145.5
Paralogidae*	Bashkirian (C)	311.7	Artinskian (P)	275.6	Kungurian (P)	270.6	311.7	311.7
Paurophlebiidae*	Carnian (Tr)	216.5	Carnian (Tr)	216.5	Norian (Tr)	203.6	216.5	216.5
Perilestidae		0.0					55.8	145.5
Permaeschnidae*	Artinskian (P)	275.6	Roadian (P)	268.0	Wordian (P)	265.8	275.6	275.6
Permagrionidae* = Permagriidae	Sakmarian (P)	284.4	Sakmarian (P)	284.4	Artinskian (P)	275.6	284.4	284.4
Permepallagidae*	Roadian (P)	268.0	Roadian (P)	268.0	Wordian (P)	265.8	275.6	275.6
Permolestidae*	Kungurian (P)	270.6	Roadian (P)	268.0	Wordian (P)	265.8	284.4	284.4
Permophlebiidae*	Wuchiapingian (P)	253.8	Wuchiapingian (P)	253.8	Changhsingian (P)	251.0	253.8	253.8
Petaluridae	Aptian (K)	112.0					155.6	155.6
Pholidoptilidae	Roadian (P)	268.0	Roadian (P)	268.0	Wordian (P)	265.8	268.0	268.0
Piroutetiidae*	Rhaetian (Tr)	199.6	Rhaetian (Tr)	199.6	Hettangian (J)	196.5	237.0	237.0
Platycnemididae	Priabonian (Pg)	33.9					33.9	112.0
Platystictidae		0.0					125.0	125.0
Polyphoridae = Polythoridae		0.0					33.9	33.9
Polytaxineuridae*	Changhsingian (P)	251.0	Changhsingian (P)	251.0	Induan (Tr)	249.5	275.6	275.6
Priscalestidae*	Lutetian (Pg)	40.4	Lutetian (Pg)	40.4	Bartonian (Pg)	37.2	40.4	40.4
Progobiaeshnidae*	Barremian (K)	125.0	Aptian (K)	112.0	Albian (K)	99.6	161.2	161.2
Prohemeroscopidae*	Tithonian (J)	145.5	Tithonian (J)	145.5	Berriasian (K)	140.2	145.5	145.5
Prostenophlebiidae*	Tithonian (J)	145.5	Tithonian (J)	145.5	Berriasian (K)	140.2	155.6	155.6
Proterogomphidae*	Tithonian (J)	145.5	Aptian (K)	112.0	Albian (K)	99.6	145.5	145.5
Protolindeniidae*	Tithonian (J)	145.5	Tithonian (J)	145.5	Berriasian (K)	140.2	155.6	161.2
Protomyrmeleontidae* = Triassagrionidae	Carnian (Tr)	216.5	Hauterivian (K)	130.0	Barremian (K)	125.0	237.0	237.0
Protoneuridae	Serravallian (Ng)	11.6					33.9	112.0
Pseudolestidae		0.0					33.9	33.9
Pseudostigmatidae		0.0					112.0	112.0
Rudiaeschnidae*	Berriasian (K)	140.2	Aptian (K)	112.0	Albian (K)	99.6	161.2	161.2
Saxonagrionidae*	Kungurian (P)	270.6	Kungurian (P)	270.6	Roadian (P)	268.0	270.6	270.6
Sieblosiidae*	Rupelian (Pg)	28.4	Tortonian (Ng)	7.2	Messinian (Ng)	5.3	48.6	48.6
Sonidae*	Hauterivian (K)	130.0	Hauterivian (K)	130.0	Barremian (K)	125.0	130.0	130.0
Sphenophlebiidae*	Toarcian (J)	175.6	Hauterivian (K)	130.0	Barremian (K)	125.0	216.5	216.5
Steleopteridae*	Oxfordian (J)	155.6	Tithonian (J)	145.5	Berriasian (K)	140.2	155.6	155.6
Stenophlebiidae*	Oxfordian (J)	155.6	Aptian (K)	112.0	Albian (K)	99.6	155.6	155.6
Synthemistidae = Synthemidae		0.0					88.6	88.6
Tarsophlebiidae*	Oxfordian (J)	155.6	Aptian (K)	112.0	Albian (K)	99.6	216.5	216.5
Triadophlebiidae*	Carnian (Tr)	216.5	Carnian (Tr)	216.5	Norian (Tr)	203.6	216.5	216.5
Triadotypidae* = Reisiidae	Anisian (Tr)	237.0	Carnian (Tr)	216.5	Norian (Tr)	203.6	237.0	237.0
Triassolestidae* = Italophlebiidae, Mesophlebiidae, Prog	Carnian (Tr)	216.5	Toarcian (J)	175.6	Aalenian (J)	171.6	216.5	216.5
Turanothemistidae* = Selenothemistidae	Toarcian (J)	175.6	Oxfordian (J)	155.6	Kimmeridgian (J)	150.8	199.6	199.6
Valdicorduliidae*	Hauterivian (K)	130.0	Hauterivian (K)	130.0	Barremian (K)	125.0	130.0	130.0
Xamenophlebiidae*	Carnian (Tr)	216.5	Toarcian (J)	175.6	Aalenian (J)	171.6	216.5	216.5
Zacallitidae*	Ypresian (Pg)	48.6	Ypresian (Pg)	48.6	Lutetian (Pg)	40.4	48.6	48.6
Zygophlebiidae*	Carnian (Tr)	216.5	Carnian (Tr)	216.5	Norian (Tr)	203.6	216.5	216.5
