# Supplementary material for: Fossil gaps inferred from phylogenies alter the apparent nature of diversification in dragonflies and their relatives
Source: BMC Evol Biol. 2011 Sep 14;11:252. doi: 10.1186/1471-2148-11-252 (PMC3179963; doi:10.1186/1471-2148-11-252)
Supplement: Additional file 5 — V and V+ scores. V and V+ support scores for MRP and MRC supertree nodes. [file 1471-2148-11-252-S5.PDF]

ADDITIONAL FILE 5 – V SCORES

Clades in both MRC and MRP trees

(Erasipteridae,Meganeuridae,Paralogidae,Namurotypidae,Permaeschnidae,Polytaxineuridae,Callimokaltaniidae,Hemizygopteridae,Ditaxineuridae,Piroutetiidae,Triadotypidae,Mitophlebiidae,Triadophlebiidae,Paurophlebiidae,Zygophlebiidae,Xamenophlebiidae,Permagrionidae,Permolestidae,Permepallagidae,Kennedyidae,Protomyrmeleontidae,Batkeniidae,Tarsophlebiidae,Turanothemistidae,Archithemistidae,Isophlebiidae,Campterophlebiidae,Sphenophlebiidae,Triassolestidae,Cyclothemistidae,Asiopteridae,Epiophlebiidae,Erichschmidtiidae,Liassophlebiidae,Juraheterophlebiidae,Heterophlebiidae,Myopophlebiidae,Liassostenophlebiidae,Gondvanogomphidae,Stenophlebiidae,Prostenophlebiidae,Henrotayiidae,Liassogomphidae,Aeschnidiidae,Cretapetaluridae,Petaluridae,Aktassiidae,Protolindeniidae,Araripegomphidae,Lindeniidae,Gomphidae,Proterogomphidae,Mesuropetalidae,Liupanshaniidae,Progobiaeshnidae,Cymatophlebiidae,Rudiaeschnidae,Paracymatophlebiidae,Aeshnidae,Eumorbaeschnidae,Austropetaliidae,Cordulegastridae,Neopetaliidae,Nannogomphidae,Hemeroscopidae,Chlorogomphidae,Araripechlorogomphidae,Juracorduliidae,Araripephlebiidae,Valdicorduliidae,Eocorduliidae,Araripelibellulidae,Synthemistidae,Cordulephyidae,Macromiidae,Corduliidae,Libellulidae,Steleopteridae,Hemiphlebiidae,Cretacoenagrionidae,Chlorolestidae,Perilestidae,Lestidae,Platystictidae,Megapodagrionidae,Hypolestidae,Isostictidae,Platycnemididae,Protoneuridae,Pseudostigmatidae,Coenagrionidae,Sieblosiidae,Pseudolestidae,Dicteriadidae,Polythoridae,Calopterygidae,Amphipterygidae,Chlorocyphidae,Zacallitidae,Epallagidae,Lestoideidae) Supporting Input Trees = 3; Conflicting Input Trees = 0; Permitting Input Trees = 0; V = 1; V+ = 1; V- = 1

(Meganeuridae,Paralogidae,Namurotypidae,Permaeschnidae,Polytaxineuridae,Callimokaltaniidae,Hemizygopteridae,Ditaxineuridae,Piroutetiidae,Triadotypidae,Mitophlebiidae,Triadophlebiidae,Paurophlebiidae,Zygophlebiidae,Xamenophlebiidae,Permagrionidae,Permolestidae,Permepallagidae,Kennedyidae,Protomyrmeleontidae,Batkeniidae,Tarsophlebiidae,Turanothemistidae,Archithemistidae,Isophlebiidae,Campterophlebiidae,Sphenophlebiidae,Triassolestidae,Cyclothemistidae,Asiopteridae,Epiophlebiidae,Erichschmidtiidae,Liassophlebiidae,Juraheterophlebiidae,Heterophlebiidae,Myopophlebiidae,Liassostenophlebiidae,Gondvanogomphidae,Stenophlebiidae,Prostenophlebiidae,Henrotayiidae,Liassogomphidae,Aeschnidiidae,Cretapetaluridae,Petaluridae,Aktassiidae,Protolindeniidae,Araripegomphidae,Lindeniidae,Gomphidae,Proterogomphidae,Mesuropetalidae,Liupanshaniidae,Progobiaeshnidae,Cymatophlebiidae,Rudiaeschnidae,Paracymatophlebiidae,Aeshnidae,Eumorbaeschnidae,Austropetaliidae,Cordulegastridae,Neopetaliidae,Nannogomphidae,Hemeroscopidae,Chlorogomphidae,Araripechlorogomphidae,Juracorduliidae,Araripephlebiidae,Valdicorduliidae,Eocorduliidae,Araripelibellulidae,Synthemistidae,Cordulephyidae,Macromiidae,Corduliidae,Libellulidae,Steleopteridae,Hemiphlebiidae,Cretacoenagrionidae,Chlorolestidae,Perilestidae,Lestidae,Platystictidae,Megapodagrionidae,Hypolestidae,Isostictidae,Platycnemididae,Protoneuridae,Pseudostigmatidae,Coenagrionidae,Sieblosiidae,Pseudolestidae,Dicteriadidae,Polythoridae,Calopterygidae,Amphipterygidae,Chlorocyphidae,Zacallitidae,Epallagidae,Lestoideidae) Supporting Input Trees = 3; Conflicting Input Trees = 0; Permitting Input Trees = 0; V = 1; V+ = 1; V- = 1

(Meganeuridae,Paralogidae,Namurotypidae) Supporting Input Trees = 5; Conflicting Input Trees = 0; Permitting Input Trees = 2; V = 1; V+ = 1; V- = 0.428571

(Meganeuridae,Paralogidae) Supporting Input Trees = 5; Conflicting Input Trees = 0; Permitting Input Trees = 2; V = 1; V+ = 1; V- = 0.428571

(Permaeschnidae,Polytaxineuridae,Callimokaltaniidae,Hemizygopteridae,Ditaxineuridae,Piroutetiidae,Triadotypidae,Mitophlebiidae,Triadophlebiidae,Paurophlebiidae,Zygophlebiidae,Xamenophlebiidae,Permagrionidae,Permolestidae,Permepallagidae,Kennedyidae,Protomyrmeleontidae,Batkeniidae,Tarsophlebiidae,Turanothemistidae,Archithemistidae,Isophlebiidae,Campterophlebiidae,Sphenophlebiidae,Triassolestidae,Cyclothemistidae,Asiopteridae,Epiophlebiidae,Erichschmidtiidae,Liassophlebiidae,Juraheterophlebiidae,Heterophlebiidae,Myopophlebiidae,Liassostenophlebiidae,Gondvanogomphidae,Stenophlebiidae,Prostenophlebiidae,Henrotayiidae,Liassogomphidae,Aeschnidiidae,Cretapetaluridae,Petaluridae,Aktassiidae,Protolindeniidae,Araripegomphidae,Lindeniidae,Gomphidae,Proterogomphidae,Mesuropetalidae,Liupanshaniidae,Progobiaeshnidae,Cymatophlebiidae,Rudiaeschnidae,Paracymatophlebiidae,Aeshnidae,Eumorbaeschnidae,Austropetaliidae,Cordulegastridae,Neopetaliidae,Nannogomphidae,Hemeroscopidae,Chlorogomphidae,Araripechlorogomphidae,Juracorduliidae,Araripephlebiidae,Valdicorduliidae,Eocorduliidae,Araripelibellulidae,Synthemistidae,Cordule

phyidae,Macromiidae,Corduliidae,Libellulidae,Steleopteridae,Hemiphlebiidae,Cretacoenagrionidae,Chlorolestidae,Perilestidae,Lestidae,Platystictidae,Megapodagrionidae,Hypolestidae,Isostictidae,Platycnemididae,Protoneuridae,Pseudostigmatidae,Coenagrionidae,Sieblosiidae,Pseudolestidae,Dicteriadidae,Polythoridae,Calopterygidae,Amphipterygidae,Chlorocyphidae,Zacallitidae,Epallagidae,Lestoideidae) Supporting Input Trees = 5; Conflicting Input Trees = 0; Permitting Input Trees = 2; V = 1; V+ = 1; V- = 0.428571

(Permaeschnidae,Polytaxineuridae,Callimokaltaniidae,Hemizygopteridae,Ditaxineuridae) Supporting Input Trees = 3; Conflicting Input Trees = 0; Permitting Input Trees = 4; V = 1; V+ = 1; V- = -0.142857

(Polytaxineuridae,Callimokaltaniidae,Hemizygopteridae,Ditaxineuridae) Supporting Input Trees = 2; Conflicting Input Trees = 1; Permitting Input Trees = 4; V = 0.333333; V+ = 0.714286; V- = -0.428571

(Callimokaltaniidae,Hemizygopteridae,Ditaxineuridae) Supporting Input Trees = 1; Conflicting Input Trees = 0; Permitting Input Trees = 6; V = 1; V+ = 1; V- = -0.714286

(Hemizygopteridae,Ditaxineuridae) Supporting Input Trees = 1; Conflicting Input Trees = 0; Permitting Input Trees = 6; V = 1; V+ = 1; V- = -0.714286

(Piroutetiidae,Triadotypidae,Mitophlebiidae,Triadophlebiidae,Paurophlebiidae,Zygophlebiidae,Xamenophlebiidae,Permagrionidae,Permolestidae,Permepallagidae,Kennedyidae,Protomyrmeleontidae,Batkeniidae,Tarsophlebiidae,Turanothemistidae,Archithemistidae,Isophlebiidae,Campterophlebiidae,Sphenophlebiidae,Triassolestidae,Cyclothemistidae,Asiopteridae,Epiophlebiidae,Erichschmidtiidae,Liassophlebiidae,Juraheterophlebiidae,Heterophlebiidae,Myopophlebiidae,Liassostenophlebiidae,Gondvanogomphidae,Stenophlebiidae,Prostenophlebiidae,Henrotayiidae,Liassogomphidae,Aeschnidiidae,Cretapetaluridae,Petaluridae,Aktassiidae,Protolindeniidae,Araripegomphidae,Lindeniidae,Gomphidae,Proterogomphidae,Mesuropetalidae,Liupanshaniidae,Progobiaeshnidae,Cymatophlebiidae,Rudiaeschnidae,Paracymatophlebiidae,Aeshnidae,Eumorbaeschnidae,Austropetaliidae,Cordulegastridae,Neopetaliidae,Nannogomphidae,Hemeroscopidae,Chlorogomphidae,Araripechlorogomphidae,Juracorduliidae,Araripephlebiidae,Valdicorduliidae,Eocorduliidae,Araripelibellulidae,Synthemistidae,Cordulephyidae,Macromiidae,Corduliidae,Libellulidae,Steleopteridae,Hemiphlebiidae,Cretacoenagrionidae,Chlorolestidae,Perilestidae,Lestidae,Platystictidae,Megapodagrionidae,Hypolestidae,Isostictidae,Platycnemididae,Protoneuridae,Pseudostigmatidae,Coenagrionidae,Sieblosiidae,Pseudolestidae,Dicteriadidae,Polythoridae,Calopterygidae,Amphipterygidae,Chlorocyphidae,Zacallitidae,Epallagidae,Lestoideidae) Supporting Input Trees = 4; Conflicting Input Trees = 2; Permitting Input Trees = 1; V = 0.333333; V+ = 0.428571; V- = 0.142857

(Piroutetiidae,Triadotypidae,Mitophlebiidae,Triadophlebiidae,Paurophlebiidae,Zygophlebiidae,Xamenophlebiidae) Supporting Input Trees = 1; Conflicting Input Trees = 0; Permitting Input Trees = 1; V = 1; V+ = 1; V- = 0

(Mitophlebiidae,Triadophlebiidae,Paurophlebiidae,Zygophlebiidae,Xamenophlebiidae) Supporting Input Trees = 1; Conflicting Input Trees = 0; Permitting Input Trees = 1; V = 1; V+ = 1; V- = 0

(Zygophlebiidae,Xamenophlebiidae) Supporting Input Trees = 1; Conflicting Input Trees = 0; Permitting Input Trees = 0; V = 1; V+ = 1; V- = 1

(Mitophlebiidae,Triadophlebiidae,Paurophlebiidae)Supporting Input Trees = 1; Conflicting Input Trees = 0; Permitting Input Trees = 1; V = 1; V+ = 1; V- = 0

(Triadophlebiidae,Paurophlebiidae) Supporting Input Trees = 1; Conflicting Input Trees = 0; Permitting Input Trees = 1; V = 1; V+ = 1; V- = 0

(Piroutetiidae,Triadotypidae) Supporting Input Trees = 1; Conflicting Input Trees = 0; Permitting Input Trees = 0; V = 1; V+ = 1; V- = 1

(Permagrionidae,Permolestidae,Permepallagidae,Kennedyidae,Protomyrmeleontidae,Batkeniidae,Tarsophlebiidae,Turanothemistidae,Archithemistidae,Isophlebiidae,Campterophlebiidae,Sphenophlebiidae,Triassolestidae,Cyclothemistidae,Asiopteridae,Epiophlebiidae,Erichschmidtiidae,Liassophlebiidae,Juraheterophlebiidae,

Heterophlebiidae,Myopophlebiidae,Liassostenophlebiidae,Gondvanogomphidae,Stenophlebiidae,Prostenophlebiidae,Henrotayiidae,Liassogomphidae,Aeschnidiidae,Cretapetaluridae,Petaluridae,Aktassiidae,Protolindeniidae,Araripegomphidae,Lindeniidae,Gomphidae,Proterogomphidae,Mesuropetalidae,Liupanshaniidae,Progobiaeshnidae,Cymatophlebiidae,Rudiaeschnidae,Paracymatophlebiidae,Aeshnidae,Eumorbaeschnidae,Austropetaliidae,Cordulegastridae,Neopetaliidae,Nannogomphidae,Hemeroscopidae,Chlorogomphidae,Araripechlorogomphidae,Juracorduliidae,Araripephlebiidae,Valdicorduliidae,Eocorduliidae,Araripelibellulidae,Synthemistidae,Cordulephyidae,Macromiidae,Corduliidae,Libellulidae,Steleopteridae,Hemiphlebiidae,Cretacoenagrionidae,Chlorolestidae,Perilestidae,Lestidae,Platystictidae,Megapodagrionidae,Hypolestidae,Isostictidae,Platycnemididae,Protoneuridae,Pseudostigmatidae,Coenagrionidae,Sieblosiidae,Pseudolestidae,Dicteriadidae,Polythoridae,Calopterygidae,Amphipterygidae,Chlorocyphidae,Zacallitidae,Epallagidae,Lestoideidae)
Supporting Input Trees = 4; Conflicting Input Trees = 3; Permitting Input Trees = 0; V = 0.142857; V+ = 0.142857; V- = 0.142857

(Permagrionidae,Permolestidae,Permepallagidae,Kennedyidae,Protomyrmeleontidae,Batkeniidae)
Supporting Input Trees = 2; Conflicting Input Trees = 1; Permitting Input Trees = 6; V = 0.333333; V+ = 0.777778; V- = -0.555556

(Permepallagidae,Kennedyidae,Protomyrmeleontidae,Batkeniidae) Supporting Input Trees = 1; Conflicting Input Trees = 1; Permitting Input Trees = 7; V = 0; V+ = 0.777778; V- = -0.777778

(Kennedyidae,Protomyrmeleontidae,Batkeniidae) Supporting Input Trees = 1; Conflicting Input Trees = 0; Permitting Input Trees = 8; V = 1; V+ = 1; V- = -0.777778

(Protomyrmeleontidae,Batkeniidae) Supporting Input Trees = 1; Conflicting Input Trees = 0; Permitting Input Trees = 4; V = 1; V+ = 1; V- = -0.6

(Permagrionidae,Permolestidae) Supporting Input Trees = 1; Conflicting Input Trees = 1; Permitting Input Trees = 1; V = 0; V+ = 0.333333; V- = -0.333333

(Tarsophlebiidae,Turanothemistidae,Archithemistidae,Isophlebiidae,Campterophlebiidae,Sphenophlebiidae,Triassolestidae,Cyclothemistidae,Asiopteridae,Epiophlebiidae,Erichschmidtiidae,Liassophlebiidae,Juraheterophlebiidae,Heterophlebiidae,Myopophlebiidae,Liassostenophlebiidae,Gondvanogomphidae,Stenophlebiidae,Prostenophlebiidae,Henrotayiidae,Liassogomphidae,Aeschnidiidae,Cretapetaluridae,Petaluridae,Aktassiidae,Protolindeniidae,Araripegomphidae,Lindeniidae,Gomphidae,Proterogomphidae,Mesuropetalidae,Liupanshaniidae,Progobiaeshnidae,Cymatophlebiidae,Rudiaeschnidae,Paracymatophlebiidae,Aeshnidae,Eumorbaeschnidae,Austropetaliidae,Cordulegastridae,Neopetaliidae,Nannogomphidae,Hemeroscopidae,Chlorogomphidae,Araripechlorogomphidae,Juracorduliidae,Araripephlebiidae,Valdicorduliidae,Eocorduliidae,Araripelibellulidae,Synthemistidae,Cordulephyidae,Macromiidae,Corduliidae,Libellulidae,Steleopteridae,Hemiphlebiidae,Cretacoenagrionidae,Chlorolestidae,Perilestidae,Lestidae,Platystictidae,Megapodagrionidae,Hypolestidae,Isostictidae,Platycnemididae,Protoneuridae,Pseudostigmatidae,Coenagrionidae,Sieblosiidae,Pseudolestidae,Dicteriadidae,Polythoridae,Calopterygidae,Amphipterygidae,Chlorocyphidae,Zacallitidae,Epallagidae,Lestoideidae) Supporting Input Trees = 5; Conflicting Input Trees = 3; Permitting Input Trees = 1; V = 0.25; V+ = 0.333333; V- = 0.111111

(Turanothemistidae,Archithemistidae,Isophlebiidae,Campterophlebiidae,Sphenophlebiidae,Triassolestidae,Cyclothemistidae,Asiopteridae,Epiophlebiidae,Erichschmidtiidae,Liassophlebiidae,Juraheterophlebiidae,Heterophlebiidae,Myopophlebiidae,Liassostenophlebiidae,Gondvanogomphidae,Stenophlebiidae,Prostenophlebiidae,Henrotayiidae,Liassogomphidae,Aeschnidiidae,Cretapetaluridae,Petaluridae,Aktassiidae,Protolindeniidae,Araripegomphidae,Lindeniidae,Gomphidae,Proterogomphidae,Mesuropetalidae,Liupanshaniidae,Progobiaeshnidae,Cymatophlebiidae,Rudiaeschnidae,Paracymatophlebiidae,Aeshnidae,Eumorbaeschnidae,Austropetaliidae,Cordulegastridae,Neopetaliidae,Nannogomphidae,Hemeroscopidae,Chlorogomphidae,Araripechlorogomphidae,Juracorduliidae,Araripephlebiidae,Valdicorduliidae,Eocorduliidae,Araripelibellulidae,Synthemistidae,Cordulephyidae,Macromiidae,Corduliidae,Libellulidae,Steleopteridae,Hemiphlebiidae,Cretacoenagrionidae,Chlorolestidae,Perilestidae,Lestidae,Platystictidae,Megapodagrionidae,Hypolestidae,Isostictidae,Platycnemididae,Protoneuridae,Pseudostigmatidae,Coenagrionidae,Sieblosiidae,Pseudolestidae,Dicteriadidae,Polythoridae,Calopterygidae,Amphipterygidae,Chlorocyphidae,Zacallitidae,Epallagidae,Lestoideidae) Supporting Input Trees = 4; Conflicting Input Trees = 5; Permitting Input Trees = 0; V = -0.111111; V+ = -0.111111; V-

= -0.111111

(Steleopteridae,Hemiphlebiidae,Cretacoenagrionidae,Chlorolestidae,Perilestidae,Lestidae,Platystictidae,Megapodagrionidae,Hypolestidae,Isostictidae,Platycnemididae,Protoneuridae,Pseudostigmatidae,Coenagrionidae,Sieblosiidae,Pseudolestidae,Dicteriadidae,Polythoridae,Calopterygidae,Amphipterygidae,Chlorocyphidae,Zacallitidae,Epallagidae,Lestoideidae) Supporting Input Trees = 16; Conflicting Input Trees = 9; Permitting Input Trees = 2; V = 0.28; V+ = 0.333333; V- = 0.185185

(Hemiphlebiidae,Cretacoenagrionidae,Chlorolestidae,Perilestidae,Lestidae,Platystictidae,Megapodagrionidae,Hypolestidae,Isostictidae,Platycnemididae,Protoneuridae,Pseudostigmatidae,Coenagrionidae,Sieblosiidae,Pseudolestidae,Dicteriadidae,Polythoridae,Calopterygidae,Amphipterygidae,Chlorocyphidae,Zacallitidae,Epallagidae,Lestoideidae) Supporting Input Trees = 15; Conflicting Input Trees = 9; Permitting Input Trees = 3; V = 0.25; V+ = 0.333333; V- = 0.111111

(Hemiphlebiidae,Cretacoenagrionidae,Chlorolestidae,Perilestidae,Lestidae) Supporting Input Trees = 7; Conflicting Input Trees = 1; Permitting Input Trees = 19; V = 0.75; V+ = 0.925926; V- = -0.481481

(Cretacoenagrionidae,Chlorolestidae,Perilestidae,Lestidae) Supporting Input Trees = 7; Conflicting Input Trees = 0; Permitting Input Trees = 20; V = 1; V+ = 1; V- = -0.481481

(Chlorolestidae,Perilestidae,Lestidae) Supporting Input Trees = 7; Conflicting Input Trees = 0; Permitting Input Trees = 20; V = 1; V+ = 1; V- = -0.481481

(Platystictidae,Megapodagrionidae,Hypolestidae,Isostictidae,Platycnemididae,Protoneuridae,Pseudostigmatidae,Coenagrionidae,Sieblosiidae,Pseudolestidae,Dicteriadidae,Polythoridae,Calopterygidae,Amphipterygidae,Chlorocyphidae,Zacallitidae,Epallagidae,Lestoideidae) Supporting Input Trees = 9; Conflicting Input Trees = 8; Permitting Input Trees = 10; V = 0.0588235; V+ = 0.407407; V- = -0.333333

(Megapodagrionidae,Hypolestidae,Isostictidae,Platycnemididae,Protoneuridae,Pseudostigmatidae,Coenagrionidae,Sieblosiidae,Pseudolestidae,Dicteriadidae,Polythoridae,Calopterygidae,Amphipterygidae,Chlorocyphidae,Zacallitidae,Epallagidae,Lestoideidae) Supporting Input Trees = 9; Conflicting Input Trees = 9; Permitting Input Trees = 9; V = 0; V+ = 0.333333; V- = -0.333333

(Megapodagrionidae,Hypolestidae,Isostictidae,Platycnemididae,Protoneuridae,Pseudostigmatidae,Coenagrionidae) Supporting Input Trees = 13; Conflicting Input Trees = 7; Permitting Input Trees = 11; V = 0.3; V+ = 0.548387; V- = -0.16129

(Isostictidae,Platycnemididae,Protoneuridae,Pseudostigmatidae,Coenagrionidae) Supporting Input Trees = 10; Conflicting Input Trees = 4; Permitting Input Trees = 17; V = 0.428571; V+ = 0.741935; V- = -0.354839

(Pseudostigmatidae,Coenagrionidae) Supporting Input Trees = 4; Conflicting Input Trees = 0; Permitting Input Trees = 28; V = 1; V+ = 1; V- = -0.75

(Isostictidae,Platycnemididae,Protoneuridae)Supporting Input Trees = 6; Conflicting Input Trees = 3; Permitting Input Trees = 11; V = 0.333333; V+ = 0.7; V- = -0.4

(Megapodagrionidae,Hypolestidae) Supporting Input Trees = 1; Conflicting Input Trees = 0; Permitting Input Trees = 9; V = 1; V+ = 1; V- = -0.8

(Sieblosiidae,Pseudolestidae,Dicteriadidae,Polythoridae,Calopterygidae,Amphipterygidae,Chlorocyphidae,Zacallitidae,Epallagidae,Lestoideidae) Supporting Input Trees = 5; Conflicting Input Trees = 7; Permitting Input Trees = 17; V = -0.166667; V+ = 0.517241; V- = -0.655172

(Pseudolestidae,Dicteriadidae,Polythoridae,Calopterygidae,Amphipterygidae,Chlorocyphidae,Zacallitidae,Epallagidae,Lestoideidae) Supporting Input Trees = 5; Conflicting Input Trees = 7; Permitting Input Trees = 17; V = -0.166667; V+ = 0.517241; V- = -0.655172

(Pseudolestidae,Dicteriadidae,Polythoridae,Calopterygidae) Supporting Input Trees = 5; Conflicting Input Trees = 5; Permitting Input Trees = 19; V = 0; V+ = 0.655172; V- = -0.655172

(Dicteriadidae,Polythoridae,Calopterygidae) Supporting Input Trees = 5; Conflicting Input Trees = 5; Permitting Input Trees = 19; V = 0; V+ = 0.655172; V- = -0.655172

(Polythoridae,Calopterygidae) Supporting Input Trees = 2; Conflicting Input Trees = 4; Permitting Input Trees = 23; V = -0.333333; V+ = 0.724138; V- = -0.862069

(Amphipterygidae,Chlorocyphidae,Zacallitidae,Epallagidae,Lestoideidae) Supporting Input Trees = 2; Conflicting Input Trees = 9; Permitting Input Trees = 2; V = -0.636364; V+ = -0.384615; V- = -0.692308

(Turanothemistidae,Archithemistidae,Isophlebiidae,Campterophlebiidae,Sphenophlebiidae,Triassolestidae,Cyclothemistidae,Asiopteridae,Epiophlebiidae,Erichschmidtiidae,Liassophlebiidae,Juraheterophlebiidae,Heterophlebiidae,Myopophlebiidae,Liassostenophlebiidae,Gondvanogomphidae,Stenophlebiidae,Prostenophlebiidae,Henrotayiidae,Liassogomphidae,Aeschnidiidae,Cretapetaluridae,Petaluridae,Aktassiidae,Protolindeniidae,Araripegomphidae,Lindeniidae,Gomphidae,Proterogomphidae,Mesuropetalidae,Liupanshaniidae,Progobiaeshnidae,Cymatophlebiidae,Rudiaeschnidae,Paracymatophlebiidae,Aeshnidae,Eumorbaeschnidae,Austropetaliidae,Cordulegastridae,Neopetaliidae,Nannogomphidae,Hemeroscopidae,Chlorogomphidae,Araripechlorogomphidae,Juracorduliidae,Araripephlebiidae,Valdicorduliidae,Eocorduliidae,Araripelibellulidae,Synthemistidae,Cordulephyidae,Macromiidae,Corduliidae,Libellulidae) Supporting Input Trees = 18; Conflicting Input Trees = 3; Permitting Input Trees = 6; V = 0.714286; V+ = 0.777778; V- = 0.333333

(Turanothemistidae,Archithemistidae,Isophlebiidae,Campterophlebiidae,Sphenophlebiidae,Triassolestidae,Cyclothemistidae,Asiopteridae) Supporting Input Trees = 3; Conflicting Input Trees = 1; Permitting Input Trees = 0; V = 0.5; V+ = 0.5; V- = 0.5

(Sphenophlebiidae,Triassolestidae,Cyclothemistidae,Asiopteridae) Supporting Input Trees = 1; Conflicting Input Trees = 0; Permitting Input Trees = 3; V = 1; V+ = 1; V- = -0.5

(Triassolestidae,Cyclothemistidae,Asiopteridae) Supporting Input Trees = 1; Conflicting Input Trees = 0; Permitting Input Trees = 3; V = 1; V+ = 1; V- = -0.5

(Triassolestidae,Cyclothemistidae) Supporting Input Trees = 1; Conflicting Input Trees = 0; Permitting Input Trees = 1; V = 1; V+ = 1; V- = 0

(Turanothemistidae,Archithemistidae,Isophlebiidae,Campterophlebiidae) Supporting Input Trees = 1; Conflicting Input Trees = 1; Permitting Input Trees = 2; V = 0; V+ = 0.5; V- = -0.5

(Archithemistidae,Isophlebiidae,Campterophlebiidae) Supporting Input Trees = 1; Conflicting Input Trees = 1; Permitting Input Trees = 2; V = 0; V+ = 0.5; V- = -0.5

(Epiophlebiidae,Erichschmidtiidae,Liassophlebiidae,Juraheterophlebiidae,Heterophlebiidae,Myopophlebiidae,Liassostenophlebiidae,Gondvanogomphidae,Stenophlebiidae,Prostenophlebiidae,Henrotayiidae,Liassogomphidae,Aeschnidiidae,Cretapetaluridae,Petaluridae,Aktassiidae,Protolindeniidae,Araripegomphidae,Lindeniidae,Gomphidae,Proterogomphidae,Mesuropetalidae,Liupanshaniidae,Progobiaeshnidae,Cymatophlebiidae,Rudiaeschnidae,Paracymatophlebiidae,Aeshnidae,Eumorbaeschnidae,Austropetaliidae,Cordulegastridae,Neopetaliidae,Nannogomphidae,Hemeroscopidae,Chlorogomphidae,Araripechlorogomphidae,Juracorduliidae,Araripephlebiidae,Valdicorduliidae,Eocorduliidae,Araripelibellulidae,Synthemistidae,Cordulephyidae,Macromiidae,Corduliidae,Libellulidae) Supporting Input Trees = 19; Conflicting Input Trees = 3; Permitting Input Trees = 5; V = 0.727273; V+ = 0.777778; V- = 0.407407

(Erichschmidtiidae,Liassophlebiidae,Juraheterophlebiidae,Heterophlebiidae,Myopophlebiidae,Liassostenophlebiidae,Gondvanogomphidae,Stenophlebiidae,Prostenophlebiidae,Henrotayiidae,Liassogomphidae,Aeschnidiidae,Cretapetaluridae,Petaluridae,Aktassiidae,Protolindeniidae,Araripegomphidae,Lindeniidae,Gomphidae,Proterogomphidae,Mesuropetalidae,Liupanshaniidae,Progobiaeshnidae,Cymatophlebiidae,Rudiaeschnidae,P

aracymatophlebiidae,Aeshnidae,Eumorbaeschnidae,Austropetaliidae,Cordulegastridae,Neopetaliidae,Nannogomphidae,Hemeroscopidae,Chlorogomphidae,Araripechlorogomphidae,Juracorduliidae,Araripephlebiidae,Valdicorduliidae,Eocorduliidae,Araripelibellulidae,Synthemistidae,Cordulephyidae,Macromiidae,Corduliidae,LibellulidaeSupporting Input Trees = 17; Conflicting Input Trees = 7; Permitting Input Trees = 5; V = 0.416667; V+ = 0.517241; V- = 0.172414

(Liassophlebiidae,Juraheterophlebiidae,Heterophlebiidae,Myopophlebiidae,Liassostenophlebiidae,Gondvanogomphidae,Stenophlebiidae,Prostenophlebiidae,Henrotayiidae,Liassogomphidae,Aeschnidiidae,Cretapetaluridae,Petaluridae,Aktassiidae,Protolindeniidae,Araripegomphidae,Lindeniidae,Gomphidae,Proterogomphidae,Mesuropetalidae,Liupanshaniidae,Progobiaeshnidae,Cymatophlebiidae,Rudiaeschnidae,Paracymatophlebiidae,Aeshnidae,Eumorbaeschnidae,Austropetaliidae,Cordulegastridae,Neopetaliidae,Nannogomphidae,Hemeroscopidae,Chlorogomphidae,Araripechlorogomphidae,Juracorduliidae,Araripephlebiidae,Valdicorduliidae,Eocorduliidae,Araripelibellulidae,Synthemistidae,Cordulephyidae,Macromiidae,Corduliidae,Libellulidae) Supporting Input Trees = 17; Conflicting Input Trees = 7; Permitting Input Trees = 5; V = 0.416667; V+ = 0.517241; V- = 0.172414

(Liassophlebiidae,Juraheterophlebiidae,Heterophlebiidae,Myopophlebiidae) Supporting Input Trees = 2; Conflicting Input Trees = 3; Permitting Input Trees = 2; V = -0.2; V+ = 0.142857; V- = -0.428571

(Liassophlebiidae,Juraheterophlebiidae,Heterophlebiidae)Supporting Input Trees = 2; Conflicting Input Trees = 3; Permitting Input Trees = 2; V = -0.2; V+ = 0.142857; V- = -0.428571

(Juraheterophlebiidae,Heterophlebiidae) Supporting Input Trees = 2; Conflicting Input Trees = 0; Permitting Input Trees = 5; V = 1; V+ = 1; V- = -0.428571

(Liassostenophlebiidae,Gondvanogomphidae,Stenophlebiidae,Prostenophlebiidae,Henrotayiidae,Liassogomphidae,Aeschnidiidae,Cretapetaluridae,Petaluridae,Aktassiidae,Protolindeniidae,Araripegomphidae,Lindeniidae,Gomphidae,Proterogomphidae,Mesuropetalidae,Liupanshaniidae,Progobiaeshnidae,Cymatophlebiidae,Rudiaeschnidae,Paracymatophlebiidae,Aeshnidae,Eumorbaeschnidae,Austropetaliidae,Cordulegastridae,Neopetaliidae,Nannogomphidae,Hemeroscopidae,Chlorogomphidae,Araripechlorogomphidae,Juracorduliidae,Araripephlebiidae,Valdicorduliidae,Eocorduliidae,Araripelibellulidae,Synthemistidae,Cordulephyidae,Macromiidae,Corduliidae,Libellulidae) Supporting Input Trees = 17; Conflicting Input Trees = 7; Permitting Input Trees = 5; V = 0.416667; V+ = 0.517241; V- = 0.172414

(Liassostenophlebiidae,Gondvanogomphidae,Stenophlebiidae,Prostenophlebiidae) Supporting Input Trees = 3; Conflicting Input Trees = 0; Permitting Input Trees = 1; V = 1; V+ = 1; V- = 0.5

(Gondvanogomphidae,Stenophlebiidae,Prostenophlebiidae) Supporting Input Trees = 3; Conflicting Input Trees = 0; Permitting Input Trees = 1; V = 1; V+ = 1; V- = 0.5

(Stenophlebiidae,Prostenophlebiidae) Supporting Input Trees = 2; Conflicting Input Trees = 0; Permitting Input Trees = 2; V = 1; V+ = 1; V- = 0

(Henrotayiidae,Liassogomphidae,Aeschnidiidae,Cretapetaluridae,Petaluridae,Aktassiidae,Protolindeniidae,Araripegomphidae,Lindeniidae,Gomphidae,Proterogomphidae,Mesuropetalidae,Liupanshaniidae,Progobiaeshnidae,Cymatophlebiidae,Rudiaeschnidae,Paracymatophlebiidae,Aeshnidae,Eumorbaeschnidae,Austropetaliidae,Cordulegastridae,Neopetaliidae,Nannogomphidae,Hemeroscopidae,Chlorogomphidae,Araripechlorogomphidae,Juracorduliidae,Araripephlebiidae,Valdicorduliidae,Eocorduliidae,Araripelibellulidae,Synthemistidae,Cordulephyidae,Macromiidae,Corduliidae,Libellulidae) Supporting Input Trees = 17; Conflicting Input Trees = 7; Permitting Input Trees = 5; V = 0.416667; V+ = 0.517241; V- = 0.172414

(Liassogomphidae,Aeschnidiidae,Cretapetaluridae,Petaluridae,Aktassiidae,Protolindeniidae,Araripegomphidae,Lindeniidae,Gomphidae,Proterogomphidae,Mesuropetalidae,Liupanshaniidae,Progobiaeshnidae,Cymatophlebiidae,Rudiaeschnidae,Paracymatophlebiidae,Aeshnidae,Eumorbaeschnidae,Austropetaliidae,Cordulegastridae,Neopetaliidae,Nannogomphidae,Hemeroscopidae,Chlorogomphidae,Araripechlorogomphidae,Juracorduliidae,Araripephlebiidae,Valdicorduliidae,Eocorduliidae,Araripelibellulidae,Synthemistidae,Cordulephyidae,Macromiidae,Corduliidae,Libellulidae) Supporting Input Trees = 17; Conflicting Input Trees = 7;

Permitting Input Trees = 5; V = 0.416667; V+ = 0.517241; V- = 0.172414

(Aeschnidiidae,Cretapetaluridae,Petaluridae,Aktassiidae,Protolindeniidae,Araripegomphidae,Lindeniidae,Gomphidae,Proterogomphidae,Mesuropetalidae,Liupanshaniidae,Progobiaeshnidae,Cymatophlebiidae,Rudiaeschnidae,Paracymatophlebiidae,Aeshnidae,Eumorbaeschnidae,Austropetaliidae,Cordulegastridae,Neopetaliidae,Nannogomphidae,Hemeroscopidae,Chlorogomphidae,Araripechlorogomphidae,Juracorduliidae,Araripephlebiidae,Valdicorduliidae,Eocorduliidae,Araripelibellulidae,Synthemistidae,Cordulephyidae,Macromiidae,Corduliidae,Libellulidae) Supporting Input Trees = 17; Conflicting Input Trees = 6; Permitting Input Trees = 6; V = 0.478261; V+ = 0.586207; V- = 0.172414

(Cretapetaluridae,Petaluridae,Aktassiidae,Protolindeniidae,Araripegomphidae,Lindeniidae,Gomphidae,Proterogomphidae,Mesuropetalidae,Liupanshaniidae,Progobiaeshnidae,Cymatophlebiidae,Rudiaeschnidae,Paracymatophlebiidae,Aeshnidae,Eumorbaeschnidae,Austropetaliidae,Cordulegastridae,Neopetaliidae,Nannogomphidae,Hemeroscopidae,Chlorogomphidae,Araripechlorogomphidae,Juracorduliidae,Araripephlebiidae,Valdicorduliidae,Eocorduliidae,Araripelibellulidae,Synthemistidae,Cordulephyidae,Macromiidae,Corduliidae,Libellulidae) Supporting Input Trees = 18; Conflicting Input Trees = 6; Permitting Input Trees = 5; V = 0.5; V+ = 0.586207; V- = 0.241379

(Cretapetaluridae,Petaluridae,Aktassiidae) Supporting Input Trees = 1; Conflicting Input Trees = 0; Permitting Input Trees = 26; V = 1; V+ = 1; V- = -0.925926

(Petaluridae,Aktassiidae) Supporting Input Trees = 1; Conflicting Input Trees = 0; Permitting Input Trees = 26; V = 1; V+ = 1; V- = -0.925926

(Araripegomphidae,Lindeniidae,Gomphidae,Proterogomphidae)Supporting Input Trees = 1; Conflicting Input Trees = 0; Permitting Input Trees = 26; V = 1; V+ = 1; V- = -0.925926

(Lindeniidae,Gomphidae,Proterogomphidae)Supporting Input Trees = 1; Conflicting Input Trees = 0; Permitting Input Trees = 26; V = 1; V+ = 1; V- = -0.925926

(Gomphidae,Proterogomphidae) Supporting Input Trees = 1; Conflicting Input Trees = 0; Permitting Input Trees = 26; V = 1; V+ = 1; V- = -0.925926

(Mesuropetalidae,Liupanshaniidae,Progobiaeshnidae,Cymatophlebiidae,Rudiaeschnidae,Paracymatophlebiidae,Aeshnidae,Eumorbaeschnidae,Austropetaliidae,Cordulegastridae,Neopetaliidae,Nannogomphidae,Hemeroscopidae,Chlorogomphidae,Araripechlorogomphidae,Juracorduliidae,Araripephlebiidae,Valdicorduliidae,Eocorduliidae,Araripelibellulidae,Synthemistidae,Cordulephyidae,Macromiidae,Corduliidae,Libellulidae)Supporting Input Trees = 8; Conflicting Input Trees = 16; Permitting Input Trees = 9; V = -0.333333; V+ = 0.030303; V- = -0.515152

(Mesuropetalidae,Liupanshaniidae,Progobiaeshnidae,Cymatophlebiidae,Rudiaeschnidae,Paracymatophlebiidae,Aeshnidae,Eumorbaeschnidae,Austropetaliidae) Supporting Input Trees = 9; Conflicting Input Trees = 0; Permitting Input Trees = 22; V = 1; V+ = 1; V- = -0.419355

(Mesuropetalidae,Liupanshaniidae) Supporting Input Trees = 2; Conflicting Input Trees = 0; Permitting Input Trees = 1; V = 1; V+ = 1; V- = 0.333333

(Progobiaeshnidae,Cymatophlebiidae,Rudiaeschnidae,Paracymatophlebiidae,Aeshnidae,Eumorbaeschnidae) Supporting Input Trees = 1; Conflicting Input Trees = 0; Permitting Input Trees = 28; V = 1; V+ = 1; V- = -0.931034

(Cymatophlebiidae,Rudiaeschnidae,Paracymatophlebiidae,Aeshnidae,Eumorbaeschnidae) Supporting Input Trees = 1; Conflicting Input Trees = 0; Permitting Input Trees = 28; V = 1; V+ = 1; V- = -0.931034

(Paracymatophlebiidae,Aeshnidae,Eumorbaeschnidae) Supporting Input Trees = 1; Conflicting Input Trees = 0; Permitting Input Trees = 26; V = 1; V+ = 1; V- = -0.925926

(Aeshnidae,Eumorbaeschnidae) Supporting Input Trees = 1; Conflicting Input Trees = 0; Permitting Input Trees = 26; V = 1; V+ = 1; V- = -0.925926

(Cymatophlebiidae,Rudiaeschnidae) Supporting Input Trees = 1; Conflicting Input Trees = 0; Permitting Input Trees = 2; V = 1; V+ = 1; V- = -0.333333

(Cordulegastridae,Neopetaliidae,Nannogomphidae,Hemeroscopidae,Chlorogomphidae,Araripechlorogomphidae,Juracorduliidae,Araripephlebiidae,Valdicorduliidae,Eocorduliidae,Araripelibellulidae,Synthemistidae,Cordulephyidae,Macromiidae,Corduliidae,Libellulidae) Supporting Input Trees = 17; Conflicting Input Trees = 9; Permitting Input Trees = 5; V = 0.307692; V+ = 0.419355; V- = 0.0967742

(Chlorogomphidae,Araripechlorogomphidae) Supporting Input Trees = 1; Conflicting Input Trees = 0; Permitting Input Trees = 17; V = 1; V+ = 1; V- = -0.888889

(Juracorduliidae,Araripephlebiidae,Valdicorduliidae,Eocorduliidae,Araripelibellulidae,Synthemistidae,Cordulephyidae,Macromiidae,Corduliidae,Libellulidae) Supporting Input Trees = 23; Conflicting Input Trees = 2; Permitting Input Trees = 10; V = 0.84; V+ = 0.885714; V- = 0.314286

(Araripephlebiidae,Valdicorduliidae,Eocorduliidae,Araripelibellulidae,Synthemistidae,Cordulephyidae,Macromiidae,Corduliidae,Libellulidae) Supporting Input Trees = 23; Conflicting Input Trees = 2; Permitting Input Trees = 10; V = 0.84; V+ = 0.885714; V- = 0.314286

(Eocorduliidae,Araripelibellulidae,Synthemistidae,Cordulephyidae,Macromiidae,Corduliidae,Libellulidae) Supporting Input Trees = 21; Conflicting Input Trees = 4; Permitting Input Trees = 10; V = 0.68; V+ = 0.771429; V- = 0.2

(Araripelibellulidae,Synthemistidae,Cordulephyidae,Macromiidae,Corduliidae,Libellulidae) Supporting Input Trees = 21; Conflicting Input Trees = 4; Permitting Input Trees = 10; V = 0.68; V+ = 0.771429; V- = 0.2

(Synthemistidae,Cordulephyidae,Macromiidae,Corduliidae,Libellulidae) Supporting Input Trees = 21; Conflicting Input Trees = 4; Permitting Input Trees = 10; V = 0.68; V+ = 0.771429; V- = 0.2

(Cordulephyidae,Macromiidae,Corduliidae,Libellulidae) Supporting Input Trees = 16; Conflicting Input Trees = 6; Permitting Input Trees = 13; V = 0.454545; V+ = 0.657143; V- = -0.0857143

(Corduliidae,Libellulidae) Supporting Input Trees = 14; Conflicting Input Trees = 9; Permitting Input Trees = 14; V = 0.217391; V+ = 0.513514; V- = -0.243243

(Cordulephyidae,Macromiidae) Supporting Input Trees = 2; Conflicting Input Trees = 0; Permitting Input Trees = 16; V = 1; V+ = 1; V- = -0.777778

(Araripephlebiidae,Valdicorduliidae) Supporting Input Trees = 1; Conflicting Input Trees = 0; Permitting Input Trees = 2; V = 1; V+ = 1; V- = -0.333333

<u>Clades in MRC tree only</u>

(Perilestidae,Lestidae) Supporting Input Trees = 4; Conflicting Input Trees = 0; Permitting Input Trees = 23; V = 1; V+ = 1; V- = -0.703704

(Platycnemididae,Protoneuridae) Supporting Input Trees = 2; Conflicting Input Trees = 0; Permitting Input Trees = 17; V = 1; V+ = 1; V- = -0.789474

(Chlorocyphidae,Zacallitidae,Epallagidae,Lestoideidae) Supporting Input Trees = 4; Conflicting Input Trees = 7; Permitting Input Trees = 2; V = -0.272727; V+ = -0.0769231; V- = -0.384615

(Zacallitidae,Epallagidae,Lestoideidae) Supporting Input Trees = 4; Conflicting Input Trees = 1; Permitting Input Trees = 8; V = 0.6; V+ = 0.846154; V- = -0.384615

(Zacallitidae,Epallagidae) Supporting Input Trees = 1; Conflicting Input Trees = 1; Permitting Input Trees = 11; V = 0; V+ = 0.846154; V- = -0.846154

(Isophlebiidae,Campterophlebiidae) Supporting Input Trees = 1; Conflicting Input Trees = 1; Permitting Input Trees = 2; V = 0; V+ = 0.5; V- = -0.5

(Cretapetaluridae,Petaluridae,Aktassiidae,Protolindeniidae) Supporting Input Trees = 1; Conflicting Input Trees = 1; Permitting Input Trees = 25; V = 0; V+ = 0.925926; V- = -0.925926

(Araripegomphidae,Lindeniidae,Gomphidae,Proterogomphidae,Mesuropetalidae,Liupanshaniidae,Progobiaeshnidae,Cymatophlebiidae,Rudiaeschnidae,Paracymatophlebiidae,Aeshnidae,Eumorbaeschnidae,Austropetaliidae,Cordulegastridae,Neopetaliidae,Nannogomphidae,Hemeroscopidae,Chlorogomphidae,Araripechlorogomphidae,Juracorduliidae,Araripephlebiidae,Valdicorduliidae,Eocorduliidae,Araripelibellulidae,Synthemistidae,Cordulephyidae,Macromiidae,Corduliidae,Libellulidae) Supporting Input Trees = 6; Conflicting Input Trees = 17; Permitting Input Trees = 10; V = -0.478261; V+ = -0.030303; V- = -0.636364

(Mesuropetalidae,Liupanshaniidae,Progobiaeshnidae,Cymatophlebiidae,Rudiaeschnidae,Paracymatophlebiidae,Aeshnidae,Eumorbaeschnidae,Austropetaliidae) Supporting Input Trees = 9; Conflicting Input Trees = 0; Permitting Input Trees = 22; V = 1; V+ = 1; V- = -0.419355

(Cordulegastridae,Neopetaliidae,Nannogomphidae,Hemeroscopidae,Chlorogomphidae,Araripechlorogomphidae,Juracorduliidae,Araripephlebiidae,Valdicorduliidae,Eocorduliidae,Araripelibellulidae,Synthemistidae,Cordulephyidae,Macromiidae,Corduliidae,Libellulidae) Supporting Input Trees = 17; Conflicting Input Trees = 9; Permitting Input Trees = 5; V = 0.307692; V+ = 0.419355; V- = 0.0967742

(Cordulegastridae,Neopetaliidae) Supporting Input Trees = 6; Conflicting Input Trees = 9; Permitting Input Trees = 11; V = -0.2; V+ = 0.307692; V- = -0.538462

(Nannogomphidae,Hemeroscopidae,Chlorogomphidae,Araripechlorogomphidae,Juracorduliidae,Araripephlebiidae,Valdicorduliidae,Eocorduliidae,Araripelibellulidae,Synthemistidae,Cordulephyidae,Macromiidae,Corduliidae,Libellulidae) Supporting Input Trees = 16; Conflicting Input Trees = 11; Permitting Input Trees = 8; V = 0.185185; V+ = 0.371429; V- = -0.0857143

(Hemeroscopidae,Chlorogomphidae,Araripechlorogomphidae,Juracorduliidae,Araripephlebiidae,Valdicorduliidae,Eocorduliidae,Araripelibellulidae,Synthemistidae,Cordulephyidae,Macromiidae,Corduliidae,Libellulidae) Supporting Input Trees = 16; Conflicting Input Trees = 11; Permitting Input Trees = 8; V = 0.185185; V+ = 0.371429; V- = -0.0857143

(Chlorogomphidae,Araripechlorogomphidae,Juracorduliidae,Araripephlebiidae,Valdicorduliidae,Eocorduliidae,Araripelibellulidae,Synthemistidae,Cordulephyidae,Macromiidae,Corduliidae,Libellulidae) Supporting Input Trees = 16; Conflicting Input Trees = 11; Permitting Input Trees = 8; V = 0.185185; V+ = 0.371429; V- = -0.0857143

Clade in MRP tree only

(Chlorolestidae,Lestidae) Supporting Input Trees = 2; Conflicting Input Trees = 0; Permitting Input Trees = 25; V = 1; V+ = 1; V- = -0.851852

(Platycnemididae,Isostictidae) Supporting Input Trees = 4; Conflicting Input Trees = 3; Permitting Input Trees = 13; V = 0.142857; V+ = 0.7; V- = -0.6

(Amphipterygidae,Chlorocyphidae,Epallagidae,Lestoideidae) Supporting Input Trees = 2; Conflicting Input Trees = 10; Permitting Input Trees = 1; V = -0.666667; V+ = -0.538462; V- = -0.692308

(Chlorocyphidae,Epallagidae,Lestoideidae) Supporting Input Trees = 4; Conflicting Input Trees = 7; Permitting Input Trees = 2; V = -0.272727; V+ = -0.0769231; V- = -0.384615

(Epallagidae,Lestoideidae) Supporting Input Trees = 4; Conflicting Input Trees = 1; Permitting Input Trees = 8; V = 0.6; V+ = 0.846154; V- = -0.384615

(Archithemistidae,Campterophlebiidae) Supporting Input Trees = 0; Conflicting Input Trees = 2; Permitting Input Trees = 0; V = -1; V+ = -1; V- = -1

(Mesuropetalidae,Liupanshaniidae,Progobiaeshnidae,Cymatophlebiidae,Rudiaeschnidae,Paracymatophlebiidae,Eumorbaeschnidae,Aeshnidae,Austropetaliidae,Hemeroscopidae,Juracorduliidae,Valdicorduliidae,Araripephlebiidae,Eocorduliidae,Araripelibellulidae,Synthemistidae,Libellulidae,Corduliidae,Cordulephyidae,Macromiidae,Cordulegastridae,Neopetaliidae,Nannogomphidae,Araripechlorogomphidae,Chlorogomphidae,Araripegomphidae,Lindeniidae,Gomphidae,Proterogomphidae,Petaluridae,Aktassiidae,Cretapetaluridae) Supporting Input Trees = 17; Conflicting Input Trees = 7; Permitting Input Trees = 5; V = 0.416667; V+ = 0.517241; V- = 0.172414

(Araripegomphidae,Lindeniidae,Gomphidae,Proterogomphidae,Petaluridae,Aktassiidae,Cretapetaluridae) Supporting Input Trees = 2; Conflicting Input Trees = 14; Permitting Input Trees = 13; V = -0.75; V+ = 0.0344828; V- = -0.862069

(Progobiaeshnidae,Cymatophlebiidae,Rudiaeschnidae,Paracymatophlebiidae,Eumorbaeschnidae,Aeshnidae, Austropetaliidae) Supporting Input Trees = 6; Conflicting Input Trees = 0; Permitting Input Trees = 25; V = 1; V+ = 1; V- = -0.612903

(Cordulegastridae,Neopetaliidae,Nannogomphidae,Araripechlorogomphidae,Chlorogomphidae) Supporting Input Trees = 4; Conflicting Input Trees = 12; Permitting Input Trees = 11; V = -0.5; V+ = 0.111111; V- = -0.703704

(Neopetaliidae,Nannogomphidae,Araripechlorogomphidae,Chlorogomphidae) Supporting Input Trees = 3; Conflicting Input Trees = 11; Permitting Input Trees = 5; V = -0.571429; V+ = -0.157895; V- = -0.684211

(Nannogomphidae,Araripechlorogomphidae,Chlorogomphidae) Supporting Input Trees = 0; Conflicting Input Trees = 1; Permitting Input Trees = 17; V = -1; V+ = 0.888889; V- = -1

(Hemeroscopidae,Juracorduliidae,Valdicorduliidae,Araripephlebiidae,Eocorduliidae,Araripelibellulidae,Synthemistidae,Libellulidae,Corduliidae,Cordulephyidae,Macromiidae) Supporting Input Trees = 22; Conflicting Input Trees = 3; Permitting Input Trees = 10; V = 0.76; V+ = 0.828571; V- = 0.257143
