# Supplementary material for: Fossil gaps inferred from phylogenies alter the apparent nature of diversification in dragonflies and their relatives
Source: BMC Evol Biol. 2011 Sep 14;11:252. doi: 10.1186/1471-2148-11-252 (PMC3179963; doi:10.1186/1471-2148-11-252)
Supplement: Additional file 6 — Sister group species richness comparisons. Sister group species richness comparisons for both MRP and MRC supertrees indicating where significant upshifts and downshifts in species richness have occurred. [file 1471-2148-11-252-S6.PDF]

ADDITIONAL FILE 6 – SISTER GROUP SPECIES RICHNESS COMPARISONS

Firstly, below is provided a list of extant family name abbreviations, which are used in the tables of sister group species richness comparisons.

Aeshnidae	Aes
Amphipterygidae	Amp
Austropetaliidae	Aus
Calopterygidae	Cal
Chlorocyphidae	Chc
Chlorogomphidae	Chg
Chlorolestidae	Chl
Cordulegastridae	Cog
Cordulephyidae	Cop
Corduliidae	Cor
Dicteriadidae	Dic
Epallagidae	Epa
Epiophlebiidae	Epi
Gomphidae	Gom
Hemiphlebiidae	Hem
Hypolestidae	Hyp
Isostictidae	Iso
Lestidae	Les
Lestoideidae	Led
Libellulidae	Lib
Lindeniidae	Lin
Macromiidae	Mac
Megapodagrionidae	Meg
Neopetaliidae	Neo
Perilestidae	Per
Petaluridae	Pet
Platycnemididae	Plc
Platystictidae	Pls
Polythoridae	Pol
Protoneuridae	Pro
Pseudolestidae	Psl
Pseudostigmatidae	Pss
Synthemistidae	Syn

Both MRC and MRP trees were considered in this analysis. When a significant difference in species richness is detected, the p value is highlighted in green. Tests are conducted as for Davis et al. (2010b). P values in light green indicate where significant upshifts in diversification are detected and p values in pink indicate significant downshifts.

<u>MRC</u>

Small Taxon	Species	Large Taxon	Species	p	
Corduliidae	244	Libellulidae	986	0.397	
Cordulephyidae	5	Macromiidae	123	0.079	
Cop,Mac	128	Lib,Cor	1230	0.189	
Synthemistidae	43	Cop,Mac,Lib,Cor	1358	0.061	
Chlorogomphidae	45	Syn,Cop,Mac,Lib,Cor	1401	0.062	
Neopetaliidae	1	Cordulegastridae	51	0.039	Sig. Diff. In SR detected
Compare with Syn,Cop,Mac,Lib,Cor clade as outgroup					
Neopetaliidae	1	Syn,Cop,Mac,Lib,Cor	1401	0.001	Sig. Diff suggests **Neopetaliidae downshift**
Cordulegastridae	51	Syn,Cop,Mac,Lib,Cor	1401	0.070	
Neo,Cog	102	Syn,Cop,Mac,Lib,Cor	1401	0.136	
Austropetaliidae	12	Aeshnidae	441	0.053	
Aus,Aes	453	Neo,Cog,Syn,Cop,Mac,Lib,Cor	1503	0.463	
Lindeniidae	32	Gomphidae	923	0.067	
Lin,Gom	955	Aus,Aes,Neo,Cog,Syn,Cop,Mac,Lib,Cor	1956	0.656	
Petaluridae	12	Lin,Gom,Aus,Aes,Neo,Cog,Syn,Cop,Mac,Lib,Cor	2911	0.008	Sig. Diff. In SR detected
Compare with Epiophlebiidae as outgroup					
Epiophlebiidae	2	Petaluridae	12	0.308	
Epiophlebiidae	2	Lin,Gom,Aus,Aes,Neo,Cog,Syn,Cop,Mac,Lib,Cor	2911	0.001	Sig. Diff suggests Li**n,Gom,Aus,Aes,Neo,Cog,Syn,Cop,Mac,Lib,Cor upshift**
Epiophlebiidae	2	Pet,Lin,Gom,Aus,Aes,Neo,Cog,Syn,Cop,Mac,Lib,Cor	24	0.160	
Lestoideidae	14	Epallagidae	69	0.341	
Led,Epa	83	Chlorocyphidae	151	0.712	
Amphipterygidae	12	Led,Epa,Chc	234	0.098	
Polythoridae	59	Calopterygidae	176	0.504	
Dicteriadidae	2	Pol,Cal	235	0.017	Sig. Diff. In SR detected
Compare with Pseudolestidae as outgroup					
Pseudolestidae	1	Dicteriadidae	2	1.000	
Pseudolestidae	1	Pol,Cal	235	0.009	Sig. Diff suggests **Pol,Cal upshift**
Pseudolestidae	1	Dic,Pol,Cal	4	0.500	
Psl,Dic,Pol,Cal	5	Amp,Led,Epa,Chc	246	0.040	Sig. Diff. In SR detected
Compare with Hyp,Meg,Coe,Pss,Iso,Pro,Plc outgroup, but need to first make SGSRCs for this clade					
Platycnemididae	227	Protoneuridae	259	0.936	
Isostictidae	45	Plc,Pro	486	0.170	

Pseudostigmatidae	19	Coenagrionidae	1121	0.033	Sig. Diff. In SR detected
Compare to Iso,Plc,Pro as outgroup					
Pseudostigmatidae	19	Iso,Plc,Pro	531	0.069	No Sig. Diff Detected
Iso,Plc,Pro	531	Coenagrionidae	1121	0.643	No Sig. Diff Detected
Iso,Plc,Pro	531	Pss,Coe	1140	0.636	
Hypolestidae	116	Megapodagrionidae	193	0.753	
Hyp,Meg	309	Iso,Plc,Pro,Pss,Coe	1671	0.312	
Use Hyp,Meg,Coe,Pss,Iso,Pro,Plc clade as outgroup for previous comparison					
Psl,Dic,Pol,Cal	5	Hyp,Meg,Coe,Pss,Iso,Pro,Plc	1980	0.005	Sig. Diff suggests **Psl,Dic,Pol,Cal downshift**
Amp,Led,Epa,Chc	246	Hyp,Meg,Coe,Pss,Iso,Pro,Plc	1980	0.221	
Psl,Dic,Pol,Cal,Amp,Led,Epa,Chc	492	Hyp,Meg,Coe,Pss,Iso,Pro,Plc	1980	0.398	
Platystictidae	214	Psl,Dic,Pol,Cal,Amp,Led,Epa,Chc,Hyp,Meg,Coe,Pss,Iso,Pro,Plc	2472	0.159	
Perilestidae	19	Lestidae	152	0.224	
Chlorolestidae	35	Per,Les	171	0.341	
Hemiphlebiidae	1	Chl,Per,Les	206	0.010	Sig. Diff. In SR detected
Compare to Pls,Psl,Dic,Pol,Cal,Amp,Led,Epa,Chc,Hyp,Meg,Coe,Pss,Iso,Pro,Plc as outgroup					
Hemiphlebiidae	1	Pls,Psl,Dic,Pol,Cal,Amp,Led,Epa,Chc,Hyp,Meg,Coe,Pss,Iso,Pro,Plc	2686	0.001	Sig. Diff suggests **Hemiphlebiidae downshift**
Chl,Per,Les	206	Pls,Psl,Dic,Pol,Cal,Amp,Led,Epa,Chc,Hyp,Meg,Coe,Pss,Iso,Pro,Plc	2686	0.143	
Hem,Chl,Per,Les	412	Pls,Psl,Dic,Pol,Cal,Amp,Led,Epa,Chc,Hyp,Meg,Coe,Pss,Iso,Pro,Plc	2686	0.266	
Epi,Pet,Lin,Gom,Aus,Aes,Neo,Cog,Syn,Cop,Mac,Lib,Cor	26	Hem,Chl,Per,Les,Pls,Psl,Dic,Pol,Cal,Amp,Led,Epa,Chc,Hyp,Meg,Coe,Pss,Iso,Pro,Plc	3098	0.017	Sig. Diff. In SR detected
Compare with Neoptera from Davis et al 2010 order-level paper – species richness value for Figure 2 tree					
Epi,Pet,Lin,Gom,Aus,Aes,Neo,Cog,Syn,Cop,Mac,Lib,Cor	26	Neoptera (Davis et al 2010a)	45400	0.001	Sig. Diff suggests E**pi,Pet,Lin,Gom,Aus,Aes,Neo,Cog,Syn,Cop,Mac,Lib,Cor downshift**
Hem,Chl,Per,Les,Pls,Psl,Dic,Pol,Cal,Amp,Led,Epa,Chc,Hyp,Meg,Coe,Pss,Iso,Pro,Plc	3098	Neoptera (Davis et al 2010a)	45400	0.128	

MRP

**Small Taxon**	**Species**	**Large Taxon**	**Species**	**p**	
Corduliidae	244	Libellulidae	986	0.397	
Cordulephyidae	5	Macromiidae	123	0.079	
Cop,Mac	128	Lib,Cor	1230	0.189	
Synthemistidae	43	Cop,Mac,Lib,Cor	1358	0.061	
Neopetaliidae	1	Chlorogomphidae	45	0.044	Sig. Diff. In SR detected
Compare with Cordulegastridae as outgroup					
Neopetaliidae	1	Cordulegastridae	51	0.039	Sig. Diff suggests **Neopetaliidae downshift**
Chlorogomphidae	45	Cordulegastridae	51	0.947	
Cordulegastridae	51	Neo,Chg	90	0.729	
Cog,Neo,Chg	141	Syn,Cop,Mac,Lib,Cor	1401	0.183	
Austropetaliidae	12	Aeshnidae	441	0.053	
Aus,Aes	453	Cog,Neo,Chg,Syn,Cop,Mac,Lib,Cor	1542	0.454	
Lindeniidae	32	Gomphidae	923	0.067	
Petaluridae	12	Lin,Gom	955	0.025	Sig. Diff. In SR detected
Compare to Aus,Aes,Cog,Neo,Chg,Syn,Cop,Mac,Lib,Cor clade as outgroup					
Petaluridae	12	Aus,Aes,Cog,Neo,Chg,Syn,Cop,Mac,Lib,Cor	1995	0.012	Sig. Diff suggests **Petaluridae downshift**
Lin,Gom	955	Aus,Aes,Cog,Neo,Chg,Syn,Cop,Mac,Lib,Cor	1995	0.648	
Pet,Lin,Gom	1910	Aus,Aes,Cog,Neo,Chg,Syn,Cop,Mac,Lib,Cor	1995	0.978	
Epiophlebiidae	2	Pet,Lin,Gom,Aus,Aes,Cog,Neo,Chg,Syn,Cop,Mac,Lib,Cor	3905	0.001	Sig. Diff. In SR detected
Compare with Hem,Per,Chl,Les,Pls,Psl,Dic,Pol,Cal,Amp,Chc,Led,Epa,Hyp,Meg,Coe,Pss,Pro,Iso,Plc clade as outgroup but need to first make SGSRCs for this clade					
Lestoideidae	14	Epallagidae	69	0.341	
Led,Epa	83	Chlorocyphidae	151	0.712	
Amphipterygidae	12	Led,Epa,Chc	234	0.098	
Polythoridae	59	Calopterygidae	176	0.504	
Dicteriadidae	2	Pol,Cal	235	0.017	Sig. Diff. In SR detected
Compare with Pseudolestidae as outgroup					
Pseudolestidae	1	Dicteriadidae	2	1.000	
Pseudolestidae	1	Pol,Cal	235	0.009	Sig. Diff suggests **Pol,Cal upshift**
Pseudolestidae	1	Dic,Pol,Cal	4	0.500	
Psl,Dic,Pol,Cal	5	Amp,Led,Epa,Chc	246	0.040	Sig. Diff. In SR detected
Compare with Hyp,Meg,Coe,Pss,Iso,Pro,Plc outgroup, but need to first make SGSRCs for this clade					
Isostictidae	45	Platycnemididae	227	0.332	
Protoneuridae	259	Iso,Plc	272	0.977	
Pseudostigmatidae	19	Coenagrionidae	1121	0.033	Sig. Diff. In SR detected
Compare to Iso,Plc,Pro as outgroup					
Pseudostigmatidae	19	Pro,Iso,Plc	531	0.069	No Sig. Diff Detected
Pro,Iso,Plc	531	Coenagrionidae	1121	0.643	No Sig. Diff Detected
Pro,Iso,Plc	531	Pss,Coe	1140	0.636	
Hypolestidae	116	Megapodagrionidae	193	0.753	
Hyp,Meg	309	Iso,Plc,Pro,Pss,Coe	1671	0.312	
Use Hyp,Meg,Coe,Pss,Iso,Pro,Plc as outgroup for previous comparison					
Psl,Dic,Pol,Cal	5	Hyp,Meg,Iso,Plc,Pro,Pss,Coe	1980	0.005	Sig. Diff suggests P**sl,Dic,Pol,Cal downshift**
Amp,Led,Epa,Chc	246	Hyp,Meg,Iso,Plc,Pro,Pss,Coe	1980	0.221	
Psl,Dic,Pol,Cal,Amp,Led,Epa,Chc	492	Hyp,Meg,Iso,Plc,Pro,Pss,Coe	1980	0.398	
Platystictidae	214	Psl,Dic,Pol,Cal,Amp,Led,Epa,Chc,Hyp,Meg,Iso,Plc,Pro,Pss,Coe	2472	0.159	
Chlorolestidae	35	Lestidae	152	0.376	
Perilestidae	19	Chl,Les	187	0.185	
Hemiphlebiidae	1	Per,Chl,Les	206	0.010	Sig. Diff. In SR detected
Compare with Pls,Psl,Dic,Pol,Cal,Amp,Led,Epa,Chc,Hyp,Meg,Iso,Plc,Pro,Pss,Coe clade as outgroup					
Hemiphlebiidae	1	Pls,Psl,Dic,Pol,Cal,Amp,Led,Epa,Chc,Hyp,Meg,Iso,Plc,Pro,Pss,Coe	2686	0.001	Sig. Diff suggests **Hemiphlebiidae downshift**
Per,Chl,Les	206	Pls,Psl,Dic,Pol,Cal,Amp,Led,Epa,Chc,Hyp,Meg,Iso,Plc,Pro,Pss,Coe	2686	0.143	
Hem,Per,Chl,Les	412	Pls,Psl,Dic,Pol,Cal,Amp,Led,Epa,Chc,Hyp,Meg,Iso,Plc,Pro,Pss,Coe	2686	0.266	
Use Hem,Per,Chl,Les,Pls,Psl,Dic,Pol,Cal,Amp,Led,Epa,Chc,Hyp,Meg,Iso,Plc,Pro,Pss,Coe clade as outgroup for previous comparison					
Epiophlebiidae	2	Hem,Per,Chl,Les,Pls,Psl,Dic,Pol,Cal,Amp,Led,Epa,Chc,Hyp,Meg,Iso,Plc,Pro,Pss,Coe	3098	0.001	Sig. Diff suggests E**piophlebiidae downshift**
Hem,Per,Chl,Les,Pls,Psl,Dic,Pol,Cal,Amp,Led,Epa,Chc,Hyp,Meg,Iso,Plc,Pro,Pss,Coe	3098	Pet,Lin,Gom,Aus,Aes,Cog,Neo,Chg,Syn,Cop,Mac,Lib,Cor	3905	0.885	
Hem,Per,Chl,Les,Pls,Psl,Dic,Pol,Cal,Amp,Led,Epa,Chc,Hyp,Meg,Iso,Plc,Pro,Pss,Coe	3098	Epi,Pet,Lin,Gom,Aus,Aes,Cog,Neo,Chg,Syn,Cop,Mac,Lib,Cor	7810	0.568	

References

Davis R. B., Baldauf S. L., Mayhew P. J. (2010a) Diversification across the hexapod orders: inferences from supertrees. *Proceedings of the Royal Society of London B* **277**: 1597-1606.

Davis R. B., Baldauf S. L., Mayhew P. J. (2010b) Diversification events in the Hymenoptera: insights from a comprehensive family-level phylogeny of the group. *BMC Evolutionary Biology* **10**: 109.
